# A Systematic Review of Direct Outputs from the Cerebellum to the Brainstem and Diencephalon in Mammals

**DOI:** 10.1007/s12311-022-01499-w

**Published:** 2022-12-28

**Authors:** Manuele Novello, Laurens W. J. Bosman, Chris I. De Zeeuw

**Affiliations:** 1 Department of Neuroscience, Erasmus MC, Rotterdam, the Netherlands; 2 Netherlands Institute for Neuroscience, Royal Academy of Arts and Sciences (KNAW), Amsterdam, the Netherlands

**Keywords:** Cerebellum, Brainstem, Diencephalon, Tracers, Rodents, Non-human primates

## Abstract

The cerebellum is involved in many motor, autonomic and cognitive functions, and new tasks that have a cerebellar contribution are discovered on a regular basis. Simultaneously, our insight into the functional compartmentalization of the cerebellum has markedly improved. Additionally, studies on cerebellar output pathways have seen a renaissance due to the development of viral tracing techniques. To create an overview of the current state of our understanding of cerebellar efferents, we undertook a systematic review of all studies on monosynaptic projections from the cerebellum to the brainstem and the diencephalon in mammals. This revealed that important projections from the cerebellum, to the motor nuclei, cerebral cortex, and basal ganglia, are predominantly di- or polysynaptic, rather than monosynaptic. Strikingly, most target areas receive cerebellar input from all three cerebellar nuclei, showing a convergence of cerebellar information at the output level. Overall, there appeared to be a large level of agreement between studies on different species as well as on the use of different types of neural tracers, making the emerging picture of the cerebellar output areas a solid one. Finally, we discuss how this cerebellar output network is affected by a range of diseases and syndromes, with also non-cerebellar diseases having impact on cerebellar output areas.

**Supplementary Information:**

The online version contains supplementary material available at 10.1007/s12311-022-01499-w.

## Introduction

Paradoxically, recent advances emphasize both heterogeneity and similarity in cerebellar function across different species of mammals. On the one hand, we now understand much better how specific tasks can be localized to certain areas of the cerebellum, in particular in relation to designated zebrin bands [1–7]. On the other hand, it is becoming clear that some cerebellar functions are more widely distributed than previously thought [8–10]. The latter may be related to a more holistic understanding of behavior. The eyeblink reflex, for instance, is not just an isolated contraction of the eyelid muscles, but part of a defensive reflex involving the whole face, or maybe even the entire body [11]. These findings are in agreement with the notion of the cerebellum as a coordinating area for most, if not all, complex motor as well as non-motor functions [5, 8, 12, 13].

**Fig. 1:**
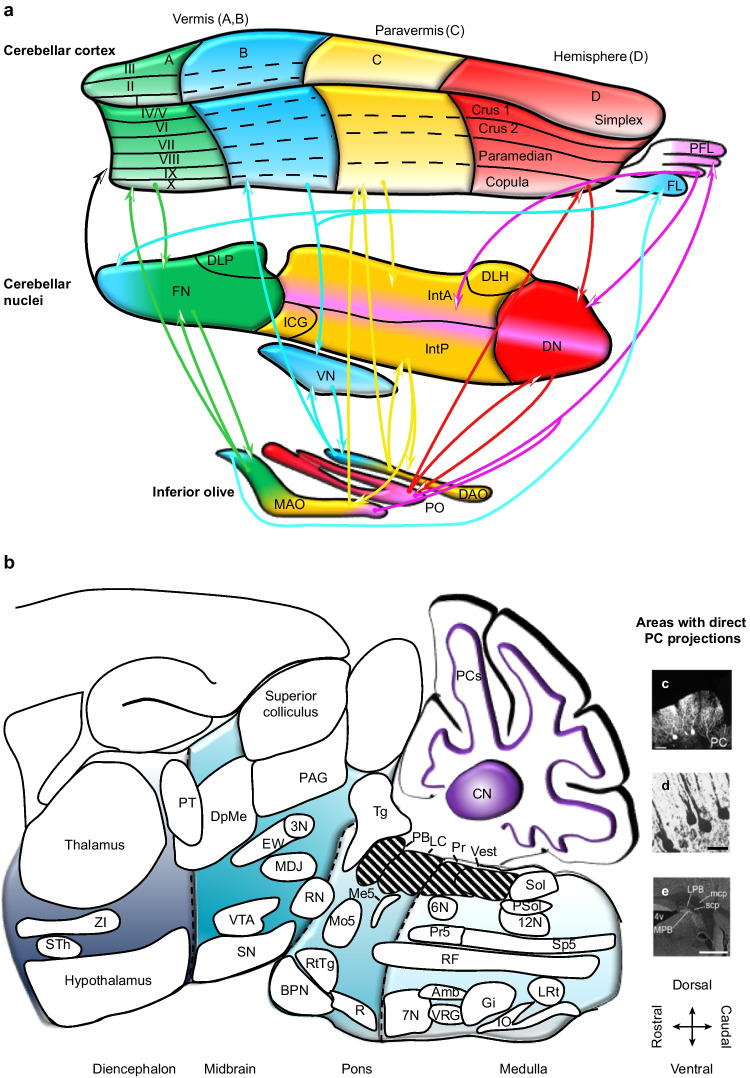
Olivocerebellar loops and Purkinje cell projections. **a** The cerebellar cortex is subdivided from medial to lateral in the vermis, paravermis, and hemispheres and from anterior to posterior in lobules I–X. In rodents, the hemispheric lobules have their own names. Lobule XI, also known as the uvula, and lobule X, sometimes called the nodulus, form together with the flocculus (FL) and the paraflocculus (PFL) the vestibulocerebellum. Purkinje cells in the cerebellar cortex project to the cerebellar nuclei, with the target area depending on the location of the Purkinje cells within the cerebellar cortex. Projections from the cerebellar nuclei to the inferior olive, and from the inferior olive to the cerebellum complement the olivocerebellar loops, organized in parasagittal modules (named A–D), as indicated by the use of different colors. Cerebellar nuclei neurons project also back to the cerebellar cortex. **b** Cerebellar output originates mostly from the cerebellar nuclei, although the striped areas receive direct input from Purkinje cells. The non-striped areas receive cerebellar input only from the cerebellar nuclei and are their locations are schematically indicated on a sagittal projection inspired by the Paxinos mouse brain atlas [39]. **c** Retrogradely labeled Purkinje cells after injection of viral tracer in the locus coeruleus (scale bar: 50 µm), reproduced with permission from [36]. The copyrights of this panel are from Nature Publishing Group. **d** Purkinje cells labeled in the ipsilateral nodulus after HRP injection into lateral vestibular nucleus (scale bar: 50 µm), reproduced from [40]. **e** Neuronal labeling into parabrachial nucleus is shown after injection of AAV-CMV-hrGFP into cerebellar lobule IX (scale bar: 1 mm), reproduced from [41]. DAO, dorsal accessory olive; DLH, dorsolateral hump; DLP, dorsolateral protuberance; DN, dentate nucleus; FN, fastigial nucleus; ICG, interstitial cell group; IntA, anterior interposed nucleus; IntP, posterior interposed nucleus; LVN, lateral vestibular nucleus; MAO, medial accessory olive; PO, principal olive; Pr, prepositus nucleus

The cerebellum is widely connected with other regions of the central nervous system [14, 15]. Ascending as well as descending input reaches the cerebellum predominantly via two glutamatergic pathways: the climbing fibers [16–18] and the mossy fibers [15, 19]. Both pathways converge in the cerebellar cortex, while also forming collaterals to the cerebellar nuclei [20]. Purkinje cells are the sole output neurons of the cerebellar cortex. They project predominantly to the cerebellar nuclei that form the main output stage of the mammalian cerebellum [20], and that form a feedback projection to the cerebellar cortex [21, 22] (Fig. 1A).

Of the cerebellar nuclei, the fastigial (or medial) nucleus is located nearest to the midline and includes also the dorsolateral protuberance and interstitial cell groups [23–27]. The fastigial nucleus receives input from Purkinje cells in the vermis [7, 20]. Lateral to the fastigial nucleus is the interposed nucleus that consists of an anterior and a posterior part [28]. Only in rodents, the anterior interposed nucleus includes also a separate region termed the dorsolateral hump [24]. The interposed nucleus receives input from Purkinje cells in the paravermis [7, 20]. The dentate (or lateral) nucleus is the most lateral cerebellar nucleus, receiving input from Purkinje cells in the hemispheres, the most lateral part of the cerebellar cortex [7, 20]. The dentate nucleus showed extreme expansion during hominid evolution, possibly related to its contribution to cognitive functions [29–31] (Fig. 1a).

In this systematic review, we summarize the available literature on cerebellar projections to the brainstem and diencephalon in mammals. We questioned whether the advent of viral tracing techniques [32–38] changed our perception of cerebellar output and whether consistent differences between species could be found. In addition, we describe whether specific diseases, whether considered to be cerebellar or non-cerebellar, are associated with modification of certain cerebellar output pathways.

## Methods

In May 2021, we searched all available entries on EMBASE, MEDLINE, Web of Science, and Cochrane published after 1975, using a query based on the terms “cerebellar efferents,” “brainstem,” and “monosynaptic” (Table S1). This resulted in 7406 references.

First, we screened the titles and abstracts of these 7406 references manually to exclude papers completely out of topic. During this round, also duplicates, studies that did not include data on mammals, and papers that were not written in English were removed. This resulted in the exclusion of 6544 references.

Of the remaining 862 references, the full text of 52 could not be retrieved through the library of the Erasmus MC, leaving a final list of 810 publications of which we screened the whole text and figures to verify the use of monosynaptic neural tracers and the documentation of the results with at least one microscopic image. The latter condition was included as we argued that the inclusion of original data is essential for the evaluation of the quality of the study. During this second round, 401 papers were selected for further study.

Finally, we read these 401 papers in detail and excluded those that did not report the use of tracers for monosynaptic cerebellar efferents. This resulted in a list of 243 papers as the basis of our systematic review. During this process, 13 new relevant studies were published and added to the final list, consisting now of 256 papers.

We scrutinized the 256 papers for reports on specific projections from the cerebellum to the brainstem and/or the diencephalon. The results are summarized in Table S2. This procedure resulted in first instance in a binary representation of brain areas: reported to receive cerebellar input or not. Table S2 is therefore primarily a resource for finding literature on putative connections. As this list potentially contains false positives, we constructed a confidence score for each monosynaptic projection. We summarized the diversity of studies that reported the existence of each pathway, using a maximum score of 10 points. To calculate the confidence score, we considered the use of both anterograde and retrograde tracers (2 points); the use of both classical and viral tracers (2 points; 1 point if multiple classical or multiple viral tracers were used, but not both); the use of multiple species (2 points for three or more species, 1 point for two species); the use of relatively large animals (2 points if cats, dogs, monkeys, or similarly large species were included as the risk for non-specific staining is less in bigger brains); and the number of replications (2 points for at least ten studies, 1 point for at least five studies) (Fig. 4).

In our summary of the literature, we also encountered studies that mentioned cerebellar projections to larger areas than the subdivisions used in our review. Whenever applicable, we mention these results at the start of the relevant sections.

Using the format of a narrative review, we included also data on the fractional anisotropy (FA) of the superior cerebellar peduncle in ataxia, schizophrenia, and autism spectrum disorder. Only papers that compared FA values of patient and control groups were included.

## Results

**Fig. 2:**
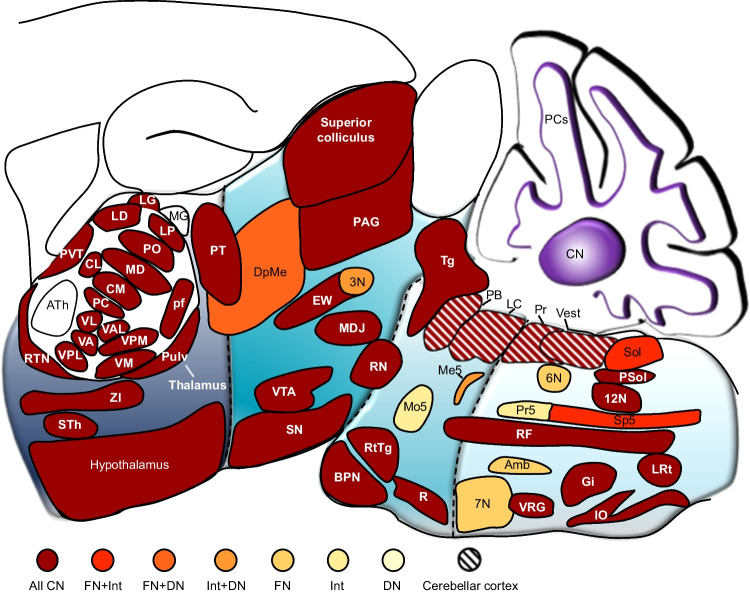
Most target areas receive input from all three cerebellar nuclei. All areas in the brainstem and diencephalon that receive monosynaptic input from the cerebellum plotted at their approximate location on a sagittal projection based on the Paxinos mouse atlas [39]. Most of these areas receive input from all three cerebellar nuclei. Abbreviations: 3 N, oculomotor nucleus; 6 N, nucleus abducens; 7 N, facial nucleus; 12 N, hypoglossal nucleus; Amb, nucleus ambiguus; CN, cerebellar nuclei; DN, dentate nucleus; DpMe, deep mesencephalic nucleus; EW, Edinger-Westphal nucleus; FN, fastigial nucleus; Gi, nucleus gigantocellularis; Int, interposed nucleus; IO, inferior olive; LC, locus coeruleus; LRt, lateral reticular nucleus; MDJ, mesodiencephalic junction; Me5, mesencephalic trigeminal nucleus; Mo5, trigeminal nucleus, motor part; PAG, periaqueductal gray; PB, parabrachial complex; Pr, prepositus nucleus; Pr5, principal trigeminal nucleus; PSol, parasolitary nucleus; PT, pretectal complex; R, raphe nuclei; RF, reticular formation; RN, red nucleus; RtTg, nucleus reticularis tegmenti pontis; SN, substantia nigra; Sol, nucleus of the solitary tract; Sp5, spinal trigeminal nucleus; STh, subthalamic nucleus; Tg, tegmentum; Vest, vestibular nuclei; VRG, ventral respiratory group; VTA, ventral tegmental area; ZI, zona incerta. Abbreviations of thalamic subnuclei: AD, antero-dorsal thalamic nucleus; AM, antero-medial thalamic nucleus; AV, antero-ventral thalamic nucleus; CL, centrolateral nucleus; CM, centromedial nucleus; IL, intralaminar nucleus; LD, laterodorsal nucleus; LG, lateral geniculate nucleus; LP, latero-posterior nucleus; MD, mediodorsal thalamic nucleus; MG, medial geniculate nucleus; PC, paracentral nucleus; pf, parafascicular complex; PO, posterior thalamic nuclei; Pulv, pulvinar nucleus; PVT, paraventricular nucleus; RTN, reticular thalamic nuclei; VA, ventro-anterior thalamic nucleus; VAL, ventro-antero thalamic complex; VL, ventrolateral thalamic nucleus; VM, ventromedial nucleus; VPL, ventro-posterior lateral thalamic nucleus; VPM, ventro-posterior medial thalamic nucleus

Cerebellar outputs target many structures of the brainstem and diencephalon. To increase readability, we describe only the main structures in the text and in Figs. 1b and 2. Further details on subnuclei can be found in Table S2.

### Projections to the Medulla Oblongata

The most caudal part of the brainstem, the medulla oblongata, is located between the spinal cord and the pons [43]. Three studies describe cerebellar pathways to the medulla oblongata without discriminating between its subdivisions [44–46].

### Medullary Reticular Formation (RF)

The medullary reticular formation consists of the gigantocellular and paragigantocellular nuclei, paramedian tract cell groups, medial reticular formation, medullary reticular nucleus, intermediate reticular nucleus, and raphe nuclei. Cerebellar pathways to the medullary reticular formation are described in eleven studies that did not discriminate between its subnuclei [32–34, 44, 47–53]. Twelve other papers describe projections to the gigantocellular nucleus [32–34, 47, 49, 51, 53–58], and four papers state the existence of cerebellar nuclear projections to the paragigantocellular nucleus [32–34, 53]. None of the studies included in this systematic review mentions projections to the paramedian tract cell groups.

The medullary raphe nuclei form a distinct division of the medullary reticular formation and consist of the obscurus, magnus, and pallidus nuclei [59, 60]. These areas contain a large proportion of serotonergic neurons and are involved in many functions such as emotion, response to stress, reward, regulation of appetite, movement, sexual behavior, sleep, respiration, and pain perception [59, 61–63]. The obscurus and magnus nuclei, but not the pallidus nucleus, receive input from the cerebellum, with all three cerebellar nuclei contributing to this output [32, 34, 55, 64–68].

### Lateral Reticular Nucleus (LRt)

The lateral reticular nucleus consists of parvocellular, magnocellular, and subtrigeminal divisions [69–72]. All output of the lateral reticular nucleus is directed to the cerebellum [73]. In return, the lateral reticular nucleus receives projections from all cerebellar nuclei. Thirteen studies describe these cerebellar efferents without taking the subdivisions of the lateral reticular nucleus into account [32, 34, 46, 47, 51, 54, 55, 67, 74–77]. Five studies describe the projections to the parvocellular part [32–34, 47] and five to the magnocellular part of the lateral reticular nucleus [32, 34, 49, 51, 54]. While the parvo- and magnocellular parts receive input from all three cerebellar nuclei, the subtrigeminal component receives only input from the fastigial nucleus [49].

### Tegmental Areas (Tg)

The tegmentum is an inner lamina of the brainstem, between the tectum and basis. The tegmentum extends along the entire length of the brainstem and is divided in two layers that both contain somatomotor and supplementary motor nuclei [78].

All cerebellar nuclei project to the pontine tegmental nuclei, ventral tegmental nucleus of Gudden, and mesopontine rostromedial tegmental nucleus [79–81]. In particular, the fastigial nucleus projects to the dorsal and medial tegmental area, to the medial pontine tegmentum, paralemniscal tegmental area, and laterodorsal tegmental and peduncular tegmental nuclei [33, 48, 79, 82]. Only the interpositus nucleus projects to the tegmental reticular nucleus [32], and only the dentate nucleus projects to the dorsomedial part of the tegmentum [83], whereas the interpositus and dentate nuclei together project to the ventral mesencephalic tegmentum and ventral midbrain tegmentum [84, 85].

### Inferior Olive (IO)

The inferior olive is located near the border of the medulla oblongata and the pons, and it consists of three subnuclei: the principal olive and the medial and dorsal accessory olives [86–88]. The caudal part of medial accessory olive further consists of subnuclei A, B, C, beta, cap of Kooy, ventrolateral protrusion, and dorsomedial cell group [89]. The inferior olive projects exclusively to the cerebellum and is the sole source of climbing fibers innervating cerebellar Purkinje cells [17, 18].

The projections from the cerebellar nuclei to the inferior olive are anatomically well-defined and largely adhere to the sagittal modules of the cerebellar cortex and the corresponding cerebellar nuclei [7]. The fastigial nucleus projects to the medial and dorsal accessory olive, the interposed nucleus to the dorsal accessory olive, and the dentate nucleus to the principal olive [32–34, 44, 54–56, 65, 67, 77, 90–121].

### Nucleus of the Solitary Tract (Sol) and Parasolitary Nucleus (PSol)

The nucleus of the solitary tract is located lateral to the motor nucleus of the vagus nerve [122, 123] and is a sensory nucleus of which the rostral part receives gustatory inputs and the caudal part cardiorespiratory input. The nucleus of the solitary tract is the main central recipient of vagal sensory input and receives signals from among others peripheral chemo-, baro-, and stretch receptors [122, 124, 125]. Immediately dorsal and lateral to the nucleus of the solitary tract is the parasolitary nucleus that is involved in the processing of vestibular and autonomic information in relation to postural control [126, 127]. It receives input from all cerebellar nuclei [34, 49, 51, 54, 56]. There are also descriptions of direct input from the fastigial and interposed nuclei to the nucleus of the solitary tract, but possibly a significant part of this input targets the parasolitary nucleus rather than the nucleus of the solitary tract itself [32, 47, 49, 55, 128–130].

### Trigeminal Nuclei (5 N)

The trigeminal nerve relates to one motor and three sensory nuclei, whereby the latter consist of a principal and a spinal nucleus in the hindbrain, as well as the mesencephalic trigeminal nucleus [131–133]. All cerebellar nuclei project to the trigeminal nuclei, but not all cerebellar nuclei target all parts of the trigeminal nuclei. The fastigial nucleus projects to the spinal trigeminal nucleus [34, 54], while the interposed nucleus projects to both motor and sensory trigeminal nuclei [32, 34]. The dentate nucleus projects to the mesencephalic trigeminal nucleus and intertrigeminal area, located between the trigeminal motor nucleus and the principal sensory nucleus [34].

### Vestibular Nuclei (Vest)

The vestibular nuclei consist of four main nuclei: the superior, medial, lateral and spinal vestibular nuclei, and some smaller structures. The spinal vestibular nucleus is also termed descending or inferior vestibular nucleus. The vestibular nuclei are located in the medulla and pons. The vestibular nuclei are involved in the control of posture, head position, equilibrium, and stabilizing vision during movement [134–136].

In contrast to most other brain areas, the vestibular nuclei receive direct input from Purkinje cells (Fig. 1d). From the flocculus, there are Purkinje cell projections to the lateral, medial, superior vestibular nuclei and to cell group Y; from the lingula to the lateral vestibular nucleus and cell group Y; from the nodulus to all vestibular nuclei; and from the uvula to the medial, spinal, and superior vestibular nuclei [40, 41, 137–152]. Furthermore, Purkinje cells in lobule III project to the lateral and inferior vestibular nuclei, and Purkinje cells of lobule IX to the superior and lateral vestibular nuclei [153]. In addition, also the three cerebellar nuclei project to all compartments of the vestibular nuclei [32–34, 40, 47, 48, 51, 52, 54–56, 64, 65, 67, 74, 93, 103, 138, 143, 149, 154, 155].

### Hypoglossal Nucleus (12 N)

The hypoglossal nucleus is located at the dorsomedial level of the medulla [156]. It houses the motor neurons of the tongue muscles and thus plays an important role in controlling swallowing, vocalization, respiration, mastication, and suckling [157–161]. Hypoglossal projections from the cerebellum were described in two papers [32, 156].

### Perihypoglossal Nucleus (Pr)

The perihypoglossal nucleus, located adjacent to the hypoglossal nucleus, consists of three subnuclei: the nucleus of Staderini, nucleus prepositus hypoglossi, and nucleus of Roller. These areas are involved in the control of eye movements [162, 163]. Cerebellar input to the perihypoglossal nucleus comes from the fastigial nucleus and targets in particular the prepositus nucleus [33, 34, 47, 54, 56, 65, 164, 165]. In addition, the prepositus nucleus receives also input from the interposed nucleus [32, 55, 67] and from Purkinje cells in the flocculus, paraflocculus, and crus I [164, 165].

### Nucleus Ambiguus (Amb)

The nucleus ambiguus is located within the medullary reticular formation and is home to vagal motor neurons projecting to upper airway muscles that play a role in respiration, swallowing and speaking, and motor neurons projecting to the cardiovascular system [166]. Although there is an early description of a fastigial projection to the nucleus ambiguus [47], this could not be reproduced later [49, 51], and we did not find other studies confirming the existence of such a projection.

### Ventral Respiratory Group (VRG)

The ventral respiratory group is located in the ventrolateral part of the medulla oblongata, and it includes, from rostral to caudal, the Bötzinger and pre-Bötzinger complexes and the rostral and caudal ventral respiratory groups [167]. The ventral respiratory group contains the central pattern generator for inspiration as well as both inspiratory and expiratory pre-motor neurons [167–169]. Only one paper describes a cerebellar nuclear projection bilaterally to the rostral ventral respiratory group [167], while we could not find evidence for cerebellar projections to other compartments of the ventral respiratory group.

#### Projections to the Metencephalon

### Pontine Reticular Formation

The pontine reticular formation is a part of the reticular formation involved in eye movement and sleep-waking cycle [170, 171]. Seven studies describe input from the cerebellum to the pontine reticular formation [33, 55, 65, 172–177], whereas ten studies state, specifically, that the paramedian reticular formation, a subdivision of the pontine reticular formation, receives projections from all cerebellar nuclei [33, 47, 49, 51, 54, 57, 178–180]. This region is represented in the figures within the medullary reticular formation (RF).

### Nucleus Reticularis Tegmenti Pontis (RtTg)

The nucleus reticularis tegmenti pontis is a specialized region within the pontine reticular formation that is best known for its involvement in oculomotor control [181–185]. Nineteen studies state that all cerebellar nuclei project to the nucleus reticularis tegmenti pontis [32, 33, 51, 55–57, 64, 65, 79, 105, 110, 117, 186–191].

### Basilar Pontine Nuclei (BPN)

The basilar pontine nuclei receive signals from among others the cerebral cortex and provide mossy fibers afferents to the cerebellum. While the basilar pontine nuclei are the main intermediate between the neocortex and the cerebellum, their functions exceed those of a mere relay station [192, 193]. During development, the size of the pontine nuclei increases together with the sizes of the cerebral and cerebellar hemispheres [192]. The basilar pontine nuclei receive projections from all three cerebellar nuclei [33, 47, 54, 65, 92, 105, 110, 117, 172, 186–188, 194–196].

### Locus Coeruleus (LC)

The locus coeruleus is located in the pontine region next to the floor of the fourth ventricle [197]. As one of the main sources of noradrenaline, it has widespread innervations serving level setting functions in modulating attention, emotion, stress, and autonomic, respiratory, sensory, as well as motor control [198, 199]. Purkinje cells, mainly from the ipsilateral medial zones, and all cerebellar nuclei, but particularly the fastigial nucleus, project to the locus coeruleus [33, 34, 36, 47, 55, 56, 176, 200, 201] (Fig. 1C).

### Parabrachial Complex (PB)

The parabrachial complex surrounds the superior cerebellar peduncle at the level of the dorsal pons. It consists of three main nuclei: the lateral and medial parabrachial nuclei that serve as alarm center, notifying the forebrain of aversive events like pain or bad taste, and the Kölliker-Fuse nucleus that is an autonomic center involved in thermoregulation and respiration [202, 203].

The parabrachial complex is rather unique in that it receives direct input from cerebellar Purkinje cells [64, 153, 204] (Fig. 1e). The medial parabrachial nucleus is targeted by Purkinje cells from lobules VIII–X [41, 144], while the lateral parabrachial nucleus receives input only from lobules IX and X [41, 144]. In addition, also all three cerebellar nuclei project to the lateral and medial parabrachial nuclei, while the rostral fastigial nucleus targets the Kölliker-Fuse nucleus as well [32–34, 49, 201, 204].

### Abducens Nucleus (6 N)

The abducens nucleus, located at the level of the pontine tegmentum and ventral to the floor of the fourth ventricle, is responsible of the movement of the eyeball in lateral direction by supplying the lateral rectus muscle [78]. Only the caudal fastigial nucleus projects to the abducens nucleus [51].

### Facial Nucleus (7 N)

The facial nucleus is located within the reticular formation at the ventrolateral part of the tegmentum of the pons and consists of motor, sensory, and parasympathetic components [78]. The motor part controls voluntary facial movement, the sensory component receives taste information, and the parasympathetic nuclei are the superior salivatory and lacrimal nuclei [78, 205]. The facial nucleus receives projections from the caudal part of fastigial nucleus and the rostral part of dorsolateral protuberance [33, 51].

#### Projections to the Mesencephalon

### Mesencephalic Reticular Formation

The mesencephalic reticular formation contains the nucleus of the posterior commissure, the central mesencephalic reticular formation, the M-group, and the rostral interstitial nucleus of the medial longitudinal fasciculus. The interstitial nucleus of Cajal belongs both to the mesencephalic reticular formation and the group of nuclei located at the mesodiencephalic junction that is discussed separately. Cerebellar projections to the mesencephalic reticular formation are described in five studies [32, 33, 44, 65, 206] and to the posterior commissure in seven studies [48, 49, 54, 67, 207–209]. This region is represented in the figures within the medullary reticular formation (RF).

### Red Nucleus (RN)

The red nucleus in the ventral midbrain consists of a caudal magnocellular and a rostral parvocellular portion and is involved in motor control, sensory processing, and higher-order cognitive functions [210, 211]. The magnocellular portion participates in organizing the execution of learned movements, coordinating the movements of extremities among quadrupedal animals, and grasping [210, 212–214], while the parvocellular portion is required for motor learning and complex cognitive-motor functions affecting the olivocerebellar system [210, 215, 216]. The parvocellular part is also considered to be involved in the group of nuclei that project to the inferior olive and that are located at the mesodiencephalic junction (see below). Both parts receive input from all cerebellar nuclei, although the magnocellular portion receives mainly input from the interposed nucleus [11, 65, 67, 77, 95, 110, 111, 217–224], and the parvocellular part predominantly from the dentate nucleus [65, 79, 110, 111, 117, 218, 220, 221, 225]. Other studies confirm these cerebello-rubral projections, although they do not take the subdivisions into account [32, 34, 48, 54, 55, 64, 83, 85, 93, 105, 121, 217, 226–245].

### Ventral Tegmental Area (VTA)

The ventral tegmental area houses dopaminergic neurons projecting to the prefrontal cortex [246–250]. It plays an important role in reward, stress, motivation, social behavior, aversion, and cognition [251–256]. The ventral tegmental area receives projections from all cerebellar nuclei [32, 37, 85, 228, 257–260].

### Mesodiencephalic Junction (MDJ)

At the mesodiencephalic junction, a group of nuclei are located that are mainly involved in oculomotor control, such as the nucleus of Darkschewitsch, medial accessory nucleus of Bechterew, parvocellular part of the red nucleus, prerubral field, rostral interstitial nucleus of the medial longitudinal fasciculus, medial accessory oculomotor nucleus, suprarubal reticular formation, nucleus of the fields of Forel, and interstitial nucleus of Cajal. The mesodiencephalic junction forms strong projections to the inferior olive and receives projections from the cerebral cortex and the cerebellum, forming an important hub in cerebro-cerebellar communication [261–272].

All three cerebellar nuclei project to the interstitial nucleus of Cajal, nucleus of Darkschewitsch, medial accessory nucleus of Bechterew, prerubral field, medial oculomotor accessory nucleus, and nucleus of fields of Forel [33, 34, 48, 54, 64, 65, 79, 83, 110, 139, 173, 180, 207, 209, 218, 230, 234, 240, 261, 273–281]. As mentioned above, cerebellar input to the parvocellular part of the red nucleus originates predominantly from the dentate nucleus [65, 79, 110, 111, 117, 218, 220, 221, 225].

### Edinger-Westphal (EW) Nucleus

The Edinger-Westphal nucleus lies dorsal to the oculomotor nucleus and controls pupillary constriction [282–285]. It receives projections from all cerebellar nuclei [34, 64, 277, 279].

### Oculomotor Nucleus (3 N)

The oculomotor nucleus is a pure motor nucleus. Together with the Edinger-Westphal nucleus, it forms the oculomotor complex involved in both somatic and autonomic functions, by innervating the muscles of the upper eyelid, eye muscles, and pupil [78, 286]. The oculomotor nucleus receives projections from the interposed and dentate nuclei, but not from the fastigial nucleus [32, 34, 230, 277].

### Pretectal Complex (PT)

Lateral to the third ventricle and caudal to the posterior thalamus, extending until the superior colliculus, one finds the pretectal complex [287]. It consists of various subnuclei, such as the pretectal olivary nucleus, posterior pretectal nucleus, medial pretectal nucleus, and anterior pretectal nucleus [288]. The pretectal complex is reciprocally connected with the Edinger-Westphal nucleus and regulates the pupillary light reflex and light-evoked blink responses and also plays a role in rapid eye movement sleep and in processing noxious stimuli [208, 287–290]. All cerebellar nuclei project to the pretectal complex [32, 48, 64, 83, 207, 208, 237, 243, 274, 291–296].

### Superior Colliculus (SC)

The superior colliculus is composed of superficial, intermediate, and deep cell layers [297]. It integrates visual, auditory, and sensorimotor information in order to orient [298–300]. The cerebellar nuclei project strongly to all layers of the superior colliculus [11, 32, 33, 44, 48, 49, 54, 65, 67, 77, 79, 83, 92, 173, 207, 208, 233, 237, 243, 275, 294, 301–311]

### Periaqueductal Gray (PAG)

The periaqueductal gray is a longitudinal gray matter structure surrounding the aqueduct of Sylvius [312–314]. It is involved in pain perception, risk assessment, responses to threats, defensive behaviors, and depression [312, 314–316]. The most rostral region of the periaqueductal gray is in proximity to the posterior commissure and to the rostral level of the third nucleus, whereas the most caudal part of the periaqueductal gray is close to the dorsal tegmental nucleus [317]. The periaqueductal gray is subdivided into four columns: the ventrolateral, lateral, dorsolateral, and dorsomedial columns [318–321]. Furthermore, the periaqueductal gray has a portion that is oculomotor-associated, termed supraoculomotor periaqueductal gray [322].

Five studies demonstrated projections from all cerebellar nuclei to the periaqueductal gray, without taking its subdivisions into account [32, 64, 65, 301, 323]. Other studies show that, in particular, the ventrolateral periaqueductal gray receives projection from all three cerebellar nuclei [32, 33, 35, 38, 48, 83, 208], the dorsolateral column from the interpositus nucleus [67, 83], the dorsomedial column from the dentate nucleus [83], and the supraoculomotor and lateral regions of periaqueductal gray from the fastigial nucleus [275].

### Deep Mesencephalic Nucleus (DpMe)

The deep mesencephalic nucleus is located between the superior colliculus, substantia nigra, red nucleus, and periaqueductal gray [324–327]. The deep mesencephalic nucleus is also termed central tegmental field, nucleus mesencephalicus profundus, midbrain reticular formation, and cuneiformis/subcuneiformis complex [328–330]. It has been suggested that the deep mesencephalic nucleus might be involved in nociception, heart rate control, arterial pressure regulation, and locomotion [331, 332]. All cerebellar nuclei project to the deep mesencephalic nucleus [33, 325].

### Substantia Nigra (SN)

The substantia nigra consists of GABA and dopaminergic neurons [333, 334] and plays a role in organizing movement, reward processing, cognitive planning, learning, and emotions [333]. All cerebellar nuclei project to the substantia nigra [33, 55, 64, 85, 260].

#### Projections to the Diencephalon

### Hypothalamus

The hypothalamus consists of several regions that manage various vital functions, such as sleep, cardiovascular regulation, stress, metabolism, thermoregulation, electrolyte and water balance, sexual behavior, feeding, and immune and endocrine responses. All these processes are linked to emotional and affective behaviors [335].

All three cerebellar nuclei project to the dorsal, posterior, lateral, ventromedial, and dorsomedial nuclei of the hypothalamus [336–343].

### Thalamus

The thalamus is the main gateway for ascending input to the cerebral cortex, while also projecting to the striatum [344, 345]. The thalamus consists of a large number of nuclei that can be organized into groups based on their location: anterior, posterior, medial, and lateral [346–348]. In this classification, the reticular nucleus has a special status as it does not project to the cerebral cortex or the striatum, but instead provides inhibition to the other thalamic nuclei [344]. In turn, the cerebral cortex projects back to the thalamus, both directly as well as indirectly via the reticular nucleus, creating cortico-thalamo-cortical connections that facilitate information exchange between different cortical areas [344, 349–352].

**Fig. 3:**
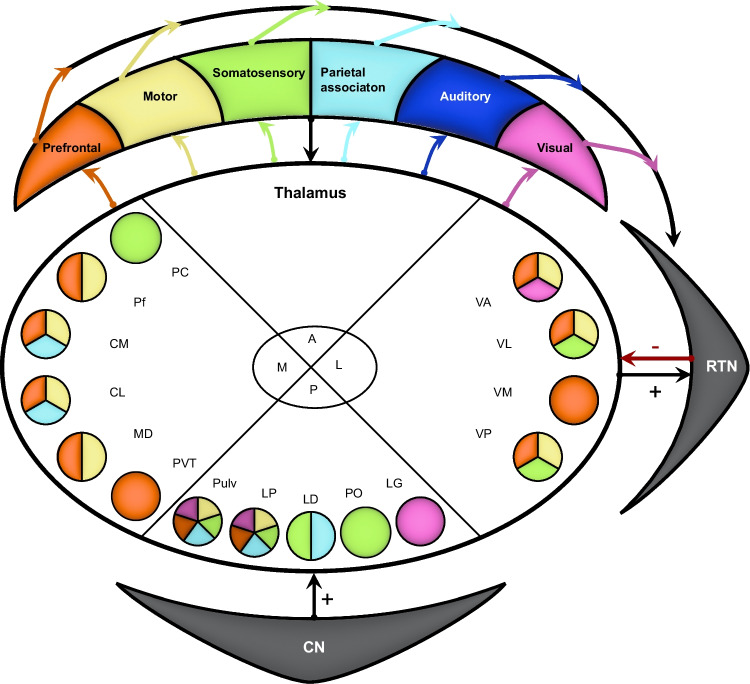
Cerebello-thalamic projections. The thalamus is an important target area of cerebellar projections and the main gateway to the cerebral cortex. The thalamic nuclei are ordered by their main target area(s) in the cerebral cortex. Most thalamic nuclei receive input from all three cerebellar nuclei. The colors of the thalamic nuclei correspond to the areas of the cerebral cortex to which they project. Abbreviations: Ath, anterior thalamic nuclei; CL, centrolateral nucleus; CM, centromedial nucleus; CN, cerebellar nuclei; IL, intralaminar nucleus; LD, laterodorsal nucleus; LG, lateral geniculate nucleus; LP, latero-posterior nucleus; MD, mediodorsal thalamic nucleus; MG, medial geniculate nucleus; PC, paracentral nucleus; Pf, parafascicular complex; PO, posterior thalamic nuclei; Pulv, pulvinar; PVT, paraventricular nucleus; RTN, reticular thalamic nuclei; VA, ventro-anterior thalamic nucleus; VL, ventrolateral thalamic nucleus; VM, ventromedial nucleus; VP, ventro-posterior thalamic nucleus

The thalamus is the main intermediate between the cerebellum and the cerebral cortex [294]. The anterior thalamus does not receive direct input from the cerebellum, but most other thalamic nuclei do. Remarkably, if a thalamic nucleus receives input from the cerebellum, it does receive input from all three cerebellar nuclei [11, 32–35, 45, 48, 52, 54, 64, 65, 67, 77, 92, 93, 104, 105, 117, 148, 175, 208, 218, 227, 230, 231, 234, 237, 240, 243, 281, 294–296, 341, 353–381] (Table S2). As different thalamic nuclei innervate different regions of the cerebral cortex [346–348], this implies that cerebellar activity has broad impact on cerebral cortical function [382] (Fig. 3).

### Subthalamic Nucleus (Sth)

The subthalamic nucleus is part of the basal ganglia and mainly involved in movement control [383]. Of note, the subthalamic nucleus is also one of the sites used for deep brain stimulation to relieve symptoms of Parkinson’s disease [384–386]. All cerebellar nuclei project to the subthalamic nucleus [64, 104, 227, 387].

### Zona Incerta (ZI)

The zona incerta is a subthalamic region with extensive projections to many areas of the brain, such as diencephalon, basal ganglia, brainstem, and spinal cord [388]. It is involved in a wide range of functions, such as visceral and arousal activities, drinking and feeding, nociception, and locomotion [389–393]. The zona incerta receives projections from all three cerebellar nuclei [32–34, 48, 64, 65, 67, 104, 218, 227, 234, 237, 240, 243, 281, 293–296, 366, 377, 381, 394–396].

## Discussion

Cerebellar projections are widespread, and there are few regions in the brainstem and diencephalon that do not receive any direct input from the cerebellum. Strikingly, most cerebellar target regions receive input from all three cerebellar nuclei. Thus, despite our increasing understanding of functional compartmentalization in the cerebellum, output streams seem to converge. This does not imply, however, that there is no heterogeneity in projections patterns within target areas or that all projections are equally strong. Based on four studies with a systematic description of multiple projections, we created a table to facilitate the comparison of projection strengths (Table 1). From Table 1, it is immediately clear that interpreting projection strength is not straightforward. It crucially depends on experimental variations and scoring techniques. A functional interpretation is even more difficult, as a weak projection to a specific nucleus can either imply a diffuse projection spreading all over that nucleus, or a targeted projection to a specific subset of neurons. For these reasons, we focus in our systematic review on the existence of evidence in favor of specific connections, rather than on the connection strengths.

**Table 1:**
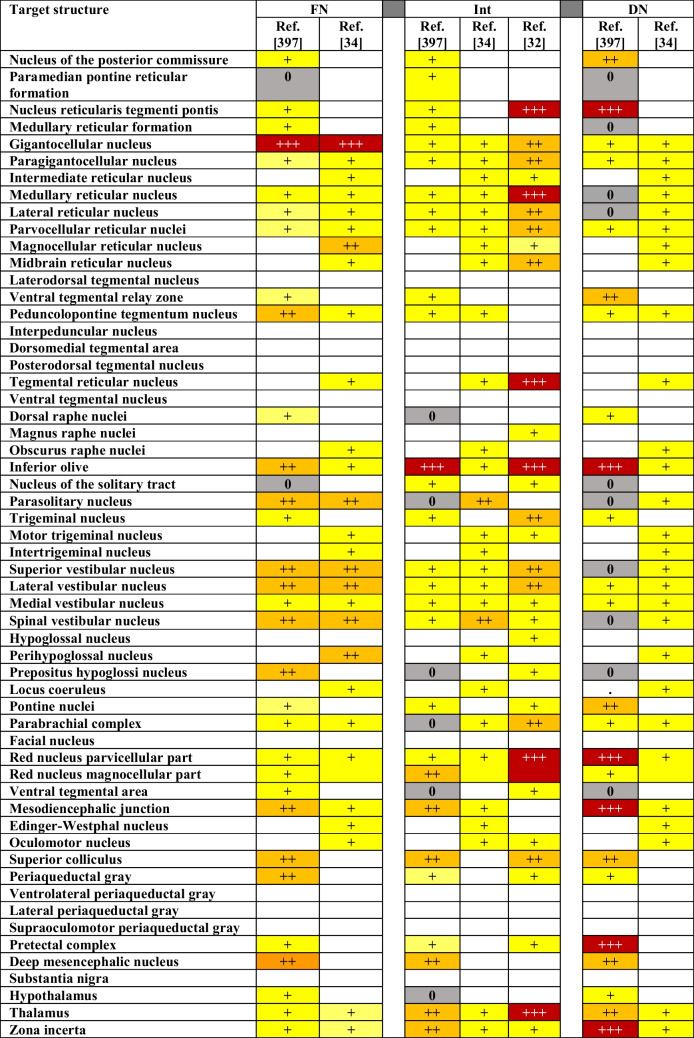
Comparing the strengths of projections. Monosynaptic projections from the fastigial, interposed and dentate nucleus to defined target regions as scored in different studies on mice (but reference [397] concerns rats). 0: reported absence of projection, + sparse projection, + + dense projection, + + + very dense projection. Blank fields concern projections that were not mentioned in a study

**Fig. 4:**
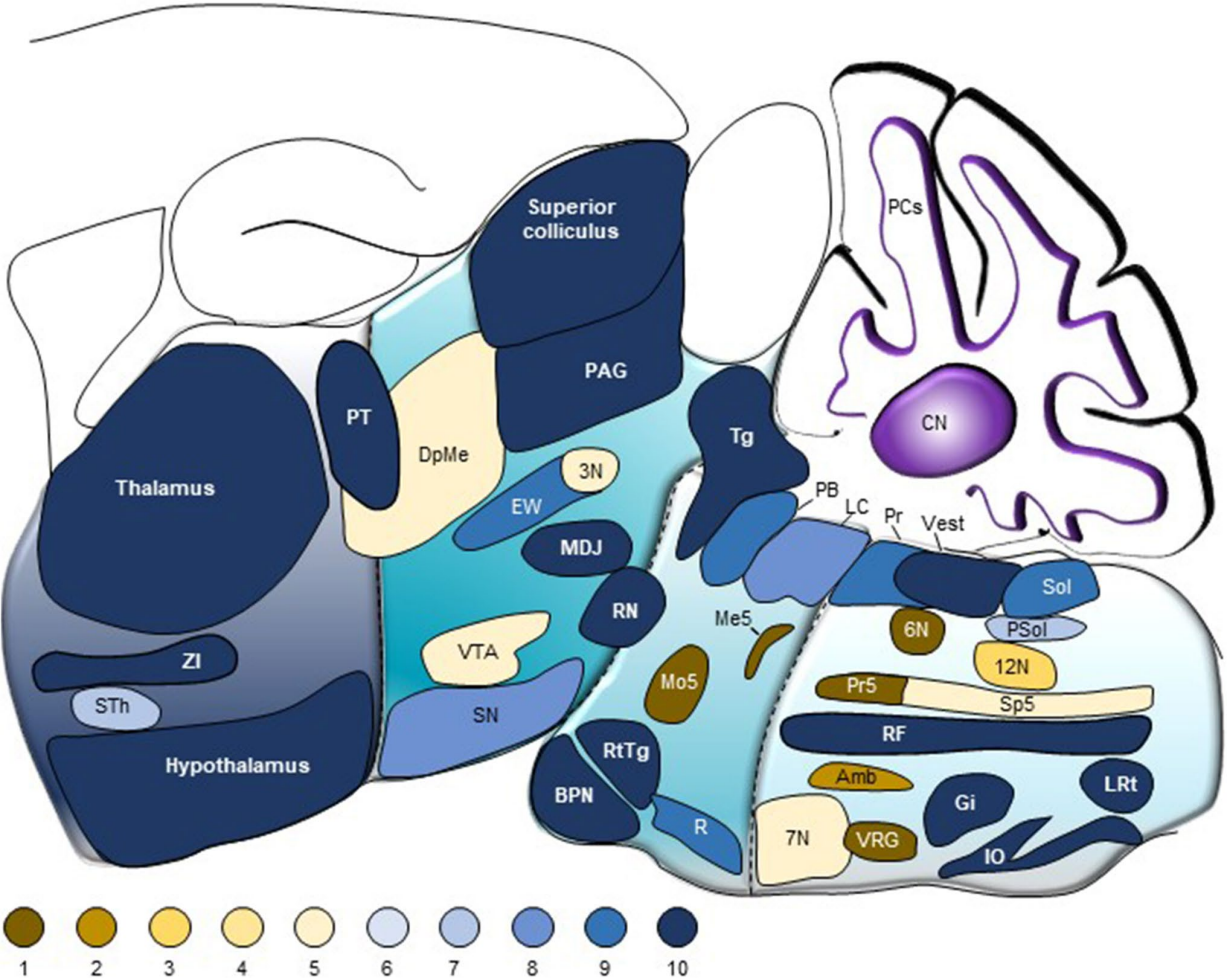
Confidence of monosynaptic projections from the cerebellum. Based on the number and types of replications, we assigned a score of 1 to 10 on the reliability of specific projections from the cerebellum (see “Methods” section). In particular, direct projections to cranial and vagal motor nuclei have been described only in single or few studies. Note that the confidence score does not make any implications on the strength of a connection (see Table 1)

While most connections have been found in multiple studies, using both antero- and retrograde tracers, utilizing classical as well as viral tracers, and examining different species, some projections have been described only in one or a few studies. As the interpretation of tracer studies can be obfuscated by non-specific staining, we consider projections described in various ways more reliable than those described only in one or a few studies. We therefore rated all projections, giving a higher confidence score to projections demonstrated under more diverse experimental conditions (Fig. 4). From this confidence rating, we noticed that cerebellar nuclei project with a high degree of confidence to the reticular formation, inferior olive, vestibular nuclei, nucleus of the solitary tract/parasolitary nucleus, locus coeruleus, parabrachial complex, tegmental area, mesodiencephalic junction, Edinger-Westphal nucleus, pontine nuclei, red nucleus, superior colliculus, periaqueductal gray, pretectal complex, thalamus, zona incerta, substantia nigra, and hypothalamus; with moderate degree of confidence to the trigeminal nucleus, facial nucleus, ventral tegmental area, oculomotor nucleus, and deep mesencephalic nucleus; and with low degree of confidence to the hypoglossal nucleus, nucleus ambiguus, nucleus abducens, and motor part of the trigeminal nucleus. It became strikingly apparent that cerebellar projections to cranial and vagal motor nuclei have only been demonstrated in relatively few publications. We consider it therefore likely that direct projections to most motor areas are either relatively sparse, or even non-existent. This argues for the dominance of indirect pathways, in a similar fashion as both the cerebral cortex and the basal ganglia are targeted disynaptically via the thalamus [354, 398]. Also, direct projections to the ventral tegmental area, the ventral respiratory group, and nucleus ambiguus have not been documented extensively, implying that also these connections could very well be either sparse or even absent. Further investigations specifically addressing the presence of these direct connections will be required to settle this issue.

### The Impact of Different Tracers

Initially, neural projections were studied by making physical or electrical lesion of the region of interest, causing degeneration of nerve terminals [400]. This method presents various complications, however, such as shrinking of cells and damage to fibers of passage [401]. Hence, we did not include studies using neurodegeneration in this systematic review.

Radioactively labelled amino acids provided more specificity than neurodegeneration, but allowed limited discrimination between labels in terminals or fibers of passage [402]. Later, horseradish peroxidase (HRP) proved to be less toxic, while providing good retrograde transport and moderate anterograde transport [403–405]. Also, HRP conjugated with wheat germ agglutinin (WGA-HRP) has been used as bidirectional tracer [401, 406, 407]. In the 1980s, *Phaseolus vulgaris*–leucoagglutinin (PHA-L) was demonstrated to be a suitable substitute for HRP as anterograde tracer [401, 408, 409]. Shortly afterwards, several other neural tracers were introduced, of which biotinylated dextran amine (BDA) [410, 411] and cholera toxin subunit B (CTb) [401, 412–415] have been used most often. All these classical tracers lack cell-type selectivity and are ill-suited for transsynaptic tracing [416, 417].

Viruses, such as adeno-associated virus (AAV) [418, 419] and herpes simplex virus [420, 421], can also be used as anterograde tracers, and rabies [422, 423], pseudorabies [424, 425], canine adenovirus [426, 427], and rAAV2-retro [428] as retrograde tracers. The specificity can be optimized by exploiting differences in cell tropism, immunogenicity, and transduction strength of different AAV strains [429, 430]. Furthermore, genetic modification of viruses can introduce conditional expression patterns so that viruses can in principle be optimized for particular experiments, including studying cell-type specific connections. In addition, also the transsynaptic properties of viral transport can be modulated. For instance, deleting external glycoprotein abolishes the ability of rabies virus to be transported transsynaptically [431, 432].

Unfortunately, even the newest conventional and viral tracers have disadvantages that can affect the interpretation of neural tracer experiments. For instance, both conventional [433–435] and viral tracers, such as canine adenovirus, may also be taken up by fibers of passage [36]. Moreover, the spreading of the conventional tracers in the injection site may lead in intense and diffuse labeling reflecting take-up by neurons or glia. This non-specific labeling may lead a unreliable identification of labeled neurons [436, 437]. In the same way, the viral tracers, due to the genome replication, may produce a strong signal, which could lead to false positive findings [438, 439]. Furthermore, different viral tracers may give different labeling results and have different neurotropism [440], creating more heterogeneity and reduced comparability between different experiments. In addition, viral tracers may cause cytotoxicity of infected neurons [416], although viral strains with lower toxicity will probably be increasingly available in the near future [440]. As each specific tracer has advantages and disadvantages, we attributed a higher confidence score to projections that have been demonstrated using both conventional and viral tracers, as well as by using anterograde and retrograde tracing (Fig. 4).

Did the introduction of viral tracers change our view of cerebellar projections? We systematically compared cerebellar projections described with classical and with viral tracers in rodents, and found more similarities than differences in the outcomes (Fig. S1). In general, viral tracers identified more targets than conventional tracers. The oculomotor, trigeminal motor, and hypoglossal nucleus did show up in experiments using viral tracers, but not in those with conventional tracers. These connections are likely to be sparse [156], and this could be an argument for the higher sensitivity of viral tracers. On the other hand, cerebellar projections to the subthalamic nucleus and the ventral respiratory group were found using conventional tracers, but not using viral tracers. Thus, both tracers confirmed the major projections of the cerebellum, but do not always agree on the sparser connections. The latter may also be due to differences in the experimental conditions.

### Similarities Between Species

Subsequently, we compared studies performed in cats, monkeys, rats or mice (Figs. S2–S5). In general, the projection patterns seemed similar. However, when examining projections from individual cerebellar nuclei, differences between species were found. A careful comparison of the study designs, however, suggests that most differences relate to variations in study design rather than to differences between species.

Nevertheless, a few exceptions remained. In particular, cerebellar projections to the nucleus abducens and nucleus ambiguus were described in cats, but not in rodents and also not in monkeys, despite searching for these areas [33, 47, 51]. Conversely, projections to the rostral ventral respiratory group, the parabrachial nucleus, the Edinger-Westphal nucleus, the ventral tegmental area, and the trigeminal nuclei were described in rodents and monkeys, but not in cats [32–34, 37, 41, 54, 64, 85, 153, 167, 228, 257–260, 277, 279, 397]. Thus, while the majority of cerebellar projections are present in monkeys, cats, and rodents, a few were not found in all species. Especially cats seem to be an outlier, which might be explained by the popularity of cats in earlier, but not in later studies. We conclude, therefore, that we did not find solid evidence for different projection patterns between mammals as divergent as mice and monkeys. We cannot exclude, however, that minor variations exist.

### Heterogeneity in Projections

Most target areas receive monosynaptic input from all three cerebellar nuclei (Fig. 2). This seemingly large convergence of cerebellar output does require some nuance, though. As indicated in the text and in Table S2, there is quite some variation of cerebellar input within certain target areas. Most brain regions are heterogeneous in terms of their neuronal cell types and projection patterns. Likewise, also the cerebellar nuclei contain multiple types of neurons [441]. Altogether, heterogeneity at the sending and the receiving side can create a complex pattern, the unraveling of which will likely entertain neuroanatomists for quite some time. As discussed above, viral tracer techniques are very helpful to study cell-type specificity.

Most projection neurons in the cerebellar nuclei are glutamatergic and they target mostly premotor areas while firing at sustained rates up to around 100 Hz [442, 443]. Excitatory neurons project also to granule and Golgi cells in the cerebellar cortex [22, 77]. Small GABAergic projection neurons target specifically the inferior olive and fire slower than glutamatergic projections neurons [442, 444, 445], but recently also other targets for inhibitory neurons were found, including sensory brainstem structures, medullary reticular nuclei, and pontine nuclei [32]. Moreover, two populations of glycinergic neurons have been found in cerebellar nuclei. One is formed by large, spontaneously active glycinergic neurons in the fastigial nucleus that target the vestibular nuclei [52], and the other is an intrinsically silent neuronal population projecting to the granular layer of the cerebellum [446]. Glycinergic neurons project also to Golgi cells in the cerebellar cortex [21]. Finally, a small population of glycinergic neurons in the fastigial nucleus project to the premotor neurons [447]. All of these cell-types of cerebellar nuclei neurons receive GABAergic projections from Purkinje cells [121, 448, 449].

Within these populations of neurons, further subdivisions can be made, as was recently studied in the fastigial nucleus. The caudal part of the fastigial nucleus expresses SNCA (alpha-synuclein), the rostral part SPP1 (osteopontin), the ventrolateral part CALB2 (calretinin), the caudal dorsolateral protuberance SPP1 and SNCA, and the rostral dorsolateral protuberance SPP1, and these subtypes contact different brainstem regions [33].

### Clinical Implications

Cerebellar degeneration, such as that occurring, for instance, in patients with types 1, 2, and 3 of spinocerebellar ataxia (SCA1, SCA2, and SCA3), can be correlated with a lower fractional anisotropy (FA) of the cerebellar peduncles [450–453]. Similarly, patients affected by Friedrich’s ataxia also presented with lower FA of dentato-rubral and dentato-thalamic tracts in the superior cerebellar peduncle [454, 455]. As the cerebellar peduncles carry all afferent and efferent fibers to and from the cerebellum [64], a lower FA could indicate reduced connectivity of the cerebellum with the rest of the brain. Indeed, there seems to be a dissociation between parietal-occipital regions and cerebellar regions in patients with SCA3 [456] and between the cerebellum and supplementary motor area, cingulate cortex, and frontal cortices in patients with Friedreich’s ataxia [457].

A reduced connectivity of cerebellum is not confined to neurodegenerative diseases classically associated with the cerebellum. For instance, also in schizophrenia, a neuropsychiatric disease characterized by hallucinations, thought disorders, social withdrawal, and cognitive dysfunction and by disrupted connectivity of large-scale neural networks [458], there is lower FA of the superior [459–461] and medial cerebellar peduncle [462–464]. This is in line with a reduced connectivity between thalamus and left and right cerebellum in patients with schizophrenia [458], as well as with hypoconnectivity of the thalamus between medial prefrontal cortex and cerebellum [465], and reduction of thalamo-prefrontal-striatal-cerebellar coupling [466]. Some connections involving the cerebellum seem, in contrast, to be stronger in patients with schizophrenia. In particular, hyperconnectivity was present between crus I and the ventral attention, motor, and auditory networks; between crus II and the ventral attention network; between lobule IX and the ventral and dorsal attention, motor, auditory, and cingulo-opercular networks; and between lobule X and the ventral attention, motor, and auditory networks [467]. This hyperconnectivity may be due to a potential association between sensorimotor lobules of the cerebellum and the cortical association networks, and use as a compensatory adaptation to maintain intrinsic baseline resting state, for instance, consciousness, processing sensory signals, and monitoring body position [467, 468].

**Fig. 5:**
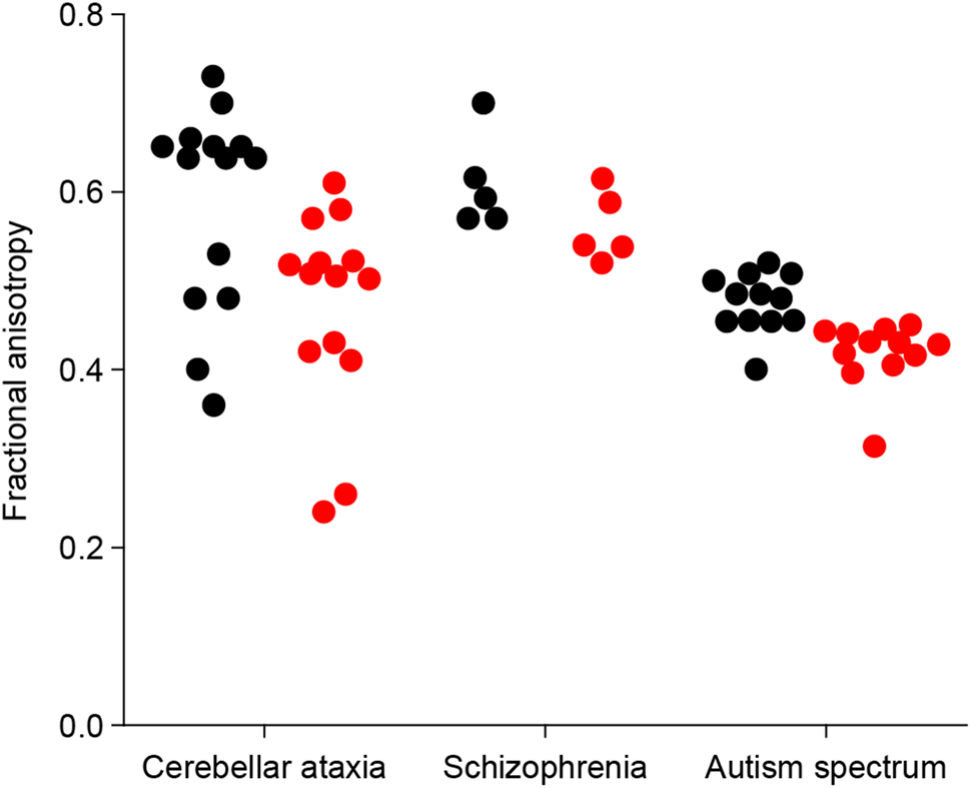
Fractional anisotropy values of superior cerebellar peduncle of patients with cerebellar ataxia, schizophrenia, and autism spectrum disorder. Fractional anisotropy values of superior cerebellar peduncle in diseases (red) and corresponding control (black) groups. Graphs showing that fractional anisotropy values of superior cerebellar peduncle in ataxia, schizophrenia, and autism spectrum disorder were significantly different than in the corresponding control groups. For details and references (see Table S3)

Finally, we examined the cerebellar functional connectivity in autism spectrum disorder (ASD), a neurodevelopmental disorder characterized by repetitive behavior, and impairments in social interaction and communication [469]. Subjects affected by ASD had decreased white matter in the dentato-rubro-thalamic tract [470] and lower FA of the superior [471, 472] and medial cerebellar peduncle [473–475]. Accordingly, patients with ASD had decreased functional connectivity between various parts of the cerebellar cortex and nuclei and different cortical regions [476–480], which may suggest an important cerebellar role in the pathophysiology of ASD. Like in schizophrenia, ASD was not only associated with reduced connectivity of particular projections, but also with increased strength of others [480–483]. In conclusion, cerebellar connectivity is affected in motor and in non-motor diseases (Fig. 5, Table S3).

## Conclusion

The cerebellum forms widespread projections, innervating most areas in the diencephalon and the brainstem, next to the spinal cord that was not part of this review. Most target areas receive input from all three cerebellar nuclei. The advent of viral tracing techniques will facilitate our understanding of the heterogeneity of these projections. Given the widespread projections, it is quite striking that most brainstem motor nuclei do not receive direct input from the cerebellum, or only sparse projections. This could be in line with modulatory and coordinating roles, rather than with direct motor control [12]. The study of these projections in diseases has just begun, but it is already clear that various diseases implicate variations in connectivity between the cerebellum and the rest of the brain.

### Supplementary Information

Below is the link to the electronic supplementary material.

Supplementary file1 (DOCX 2.98 MB)

## Data Availability

Not applicable.
