# Correction to: A Systematic Review of Direct Outputs from the Cerebellum to the Brainstem and Diencephalon in Mammals

**DOI:** 10.1007/s12311-023-01566-w

**Published:** 2023-05-12

**Authors:** Manuele Novello, Laurens W. J. Bosman, Chris I. De Zeeuw

**Affiliations:** 1 Department of Neuroscience, Erasmus MC, Rotterdam, the Netherlands; 2 Royal Academy of Arts and Sciences (KNAW), Netherlands Institute for Neuroscience, Amsterdam, the Netherlands

**Correction to: The Cerebellum**

**https://doi.org/10.1007/s12311-022-01499-w**

The original version of this article unfortunately contained a mistake.

The references cited in Table 1 were not adjusted during the proofing stage. Reference 398 should be listed as Reference 397. Also, the table has been simplified, as shown below.

**Table 1:**
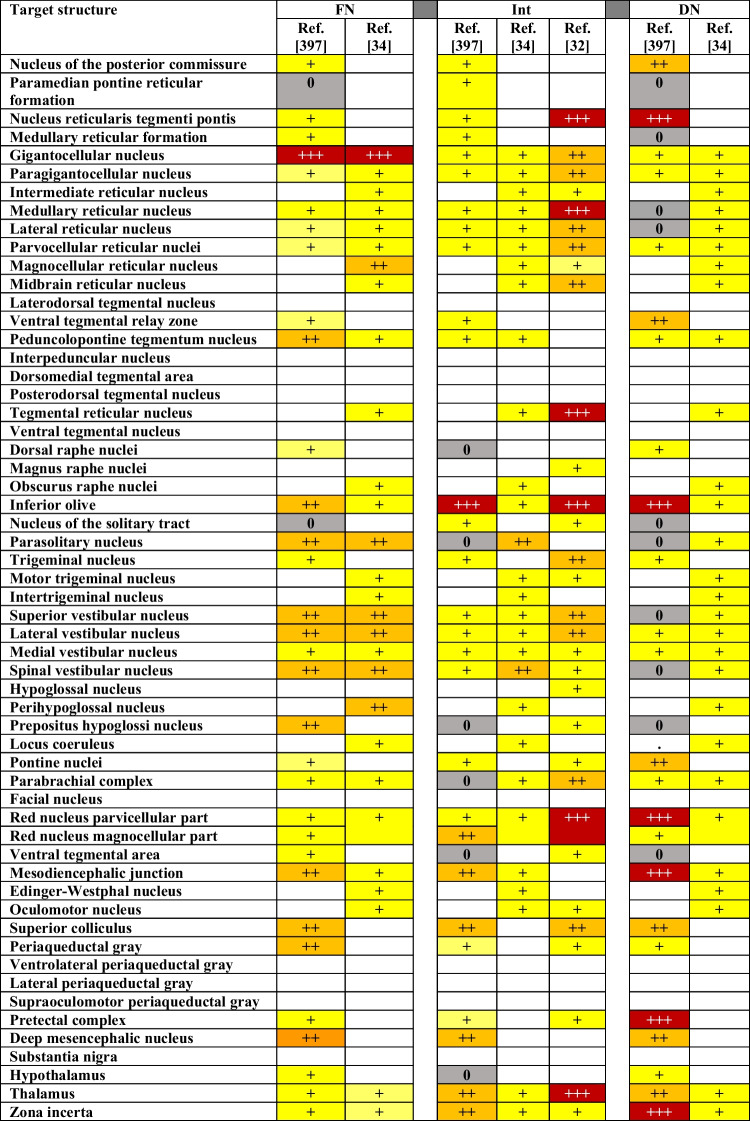
Comparing the strengths of projections

*Monosynaptic projections from the fastigial, interposed and dentate nucleus to defined target regions as scored in different studies on mice (but reference [397] concerns rats). 0: reported absence of projection,* + *sparse projection,* +  + *dense projection,* +  +  + *very dense projection. Blank fields concern projections that were not mentioned in a study*

The original article has been corrected.

