# Supplementary material for: A Systematic Review of Direct Outputs from the Cerebellum to the Brainstem and Diencephalon in Mammals
Source: Cerebellum. 2022 Dec 28;23(1):210–39. doi: 10.1007/s12311-022-01499-w (PMC10864519; doi:10.1007/s12311-022-01499-w)
Supplement: Supplementary file 1 — Supplementary file1 (DOCX 2.98 MB) [file 12311_2022_1499_MOESM1_ESM.docx]

***
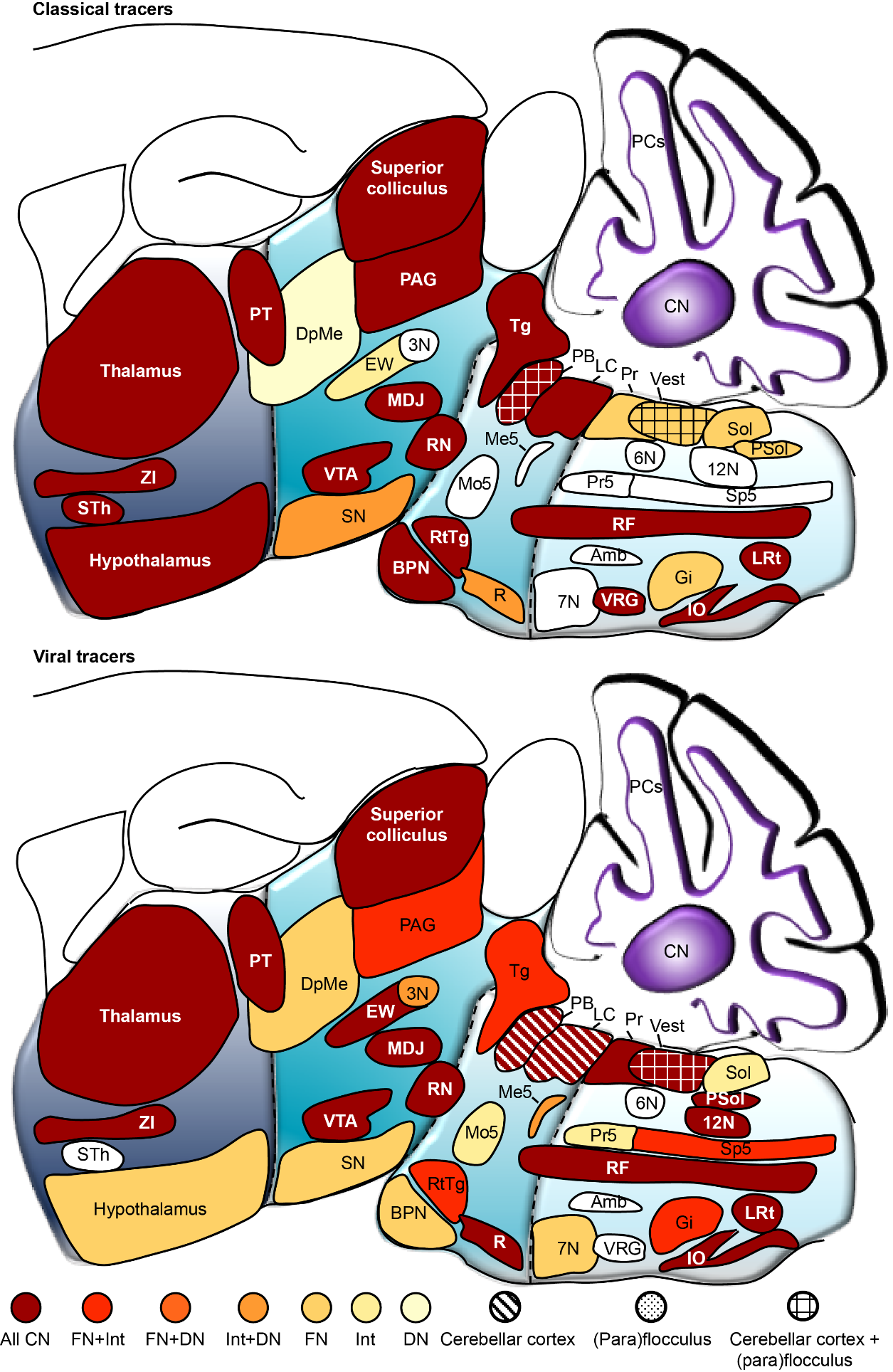
Figure S1. Cerebellar projection patterns using classical vs. viral tracers in rodents.***

*Sagittal templates of mouse brain from Paxinos’ atlas [*39*] showing brain regions colored according to the cerebellar projection that they receive. The comparison of the cerebellar projection pattern using classical (top) and viral (bottom) tracers in rodents.*

***
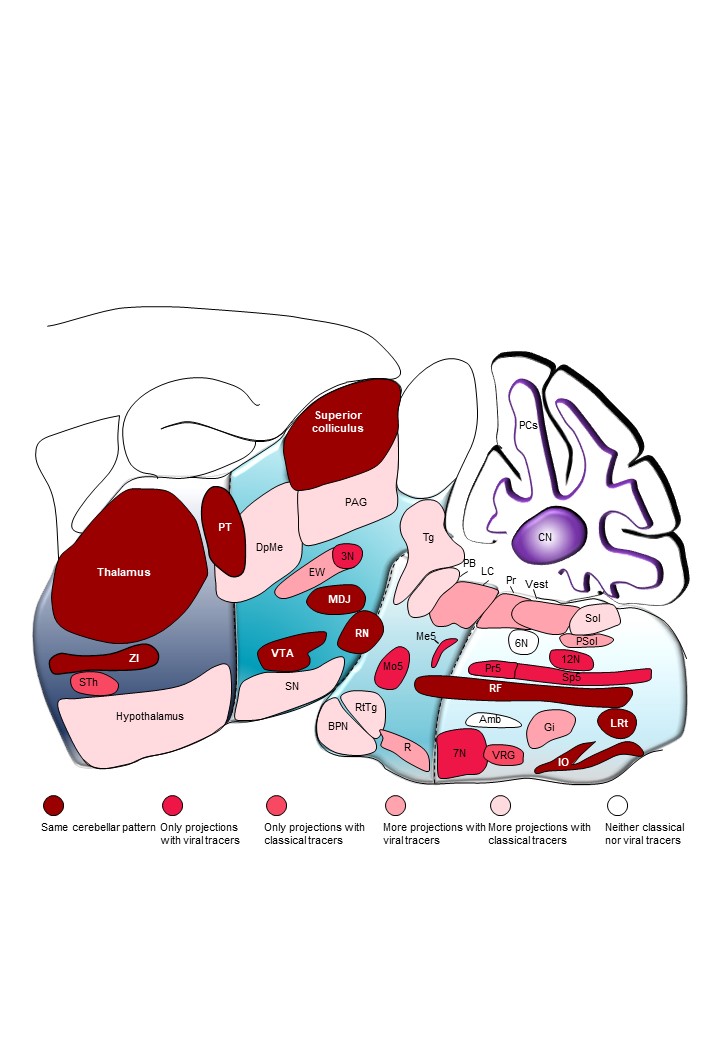
***

***Figure S2. Differences and similarities of cerebellar projection patterns using classical vs. viral tracers.***

*Sagittal templates of mouse brain from Paxinos’ atlas [*39*] showing brain regions colored according to the cerebellar projection that they receive using classical and viral tracers in rodents.*

***
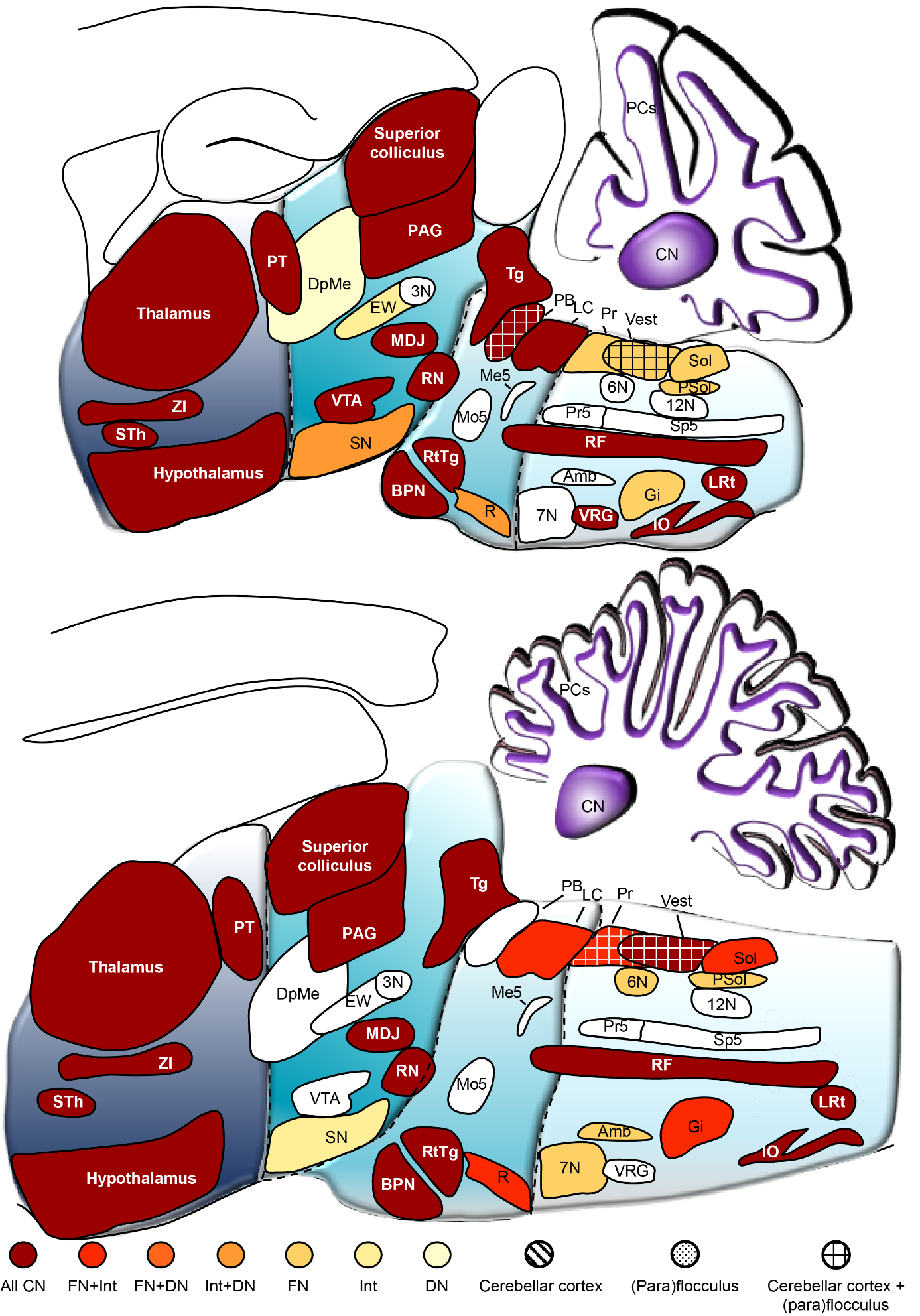
***

***Figure S3. Comparison of cerebellar projection patterns in rodents vs. cats.***

*As figure S1. The comparison of the cerebellar projection pattern between rodents (top) and cats (bottom) is shown, with the use of classical tracers.*

***
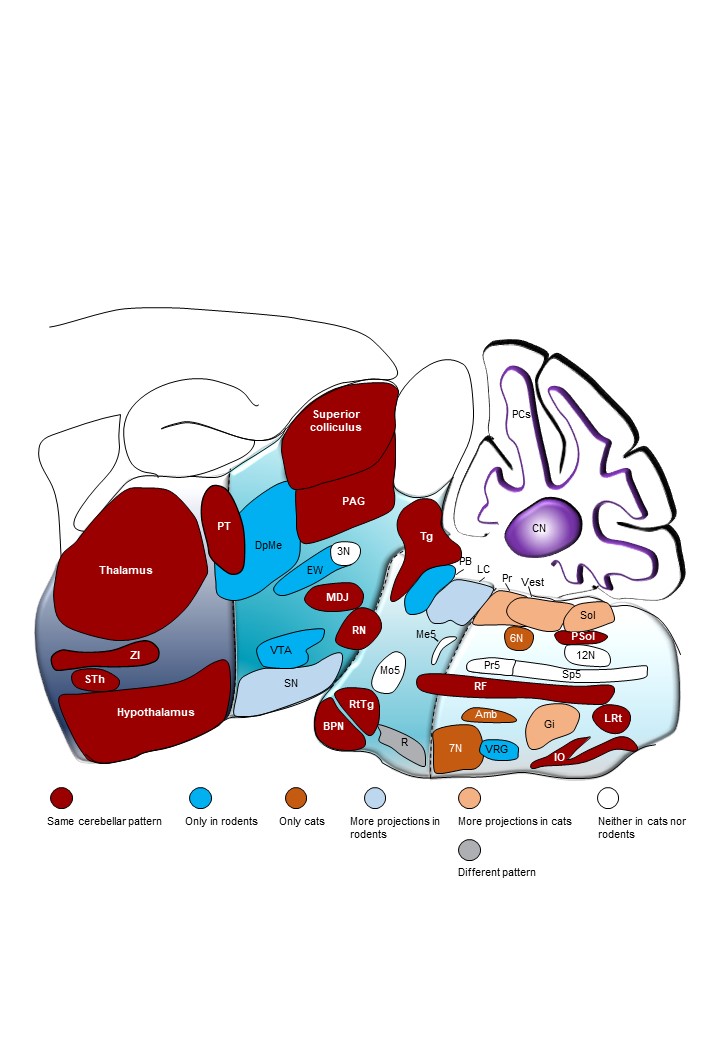
***

***Figure S4. Differences and similarities in rodents vs. cats using classical tracers.***

*As figure S2, but showing brain regions colored according to the cerebellar projection that they receive in cats and rodents using classical tracers.*


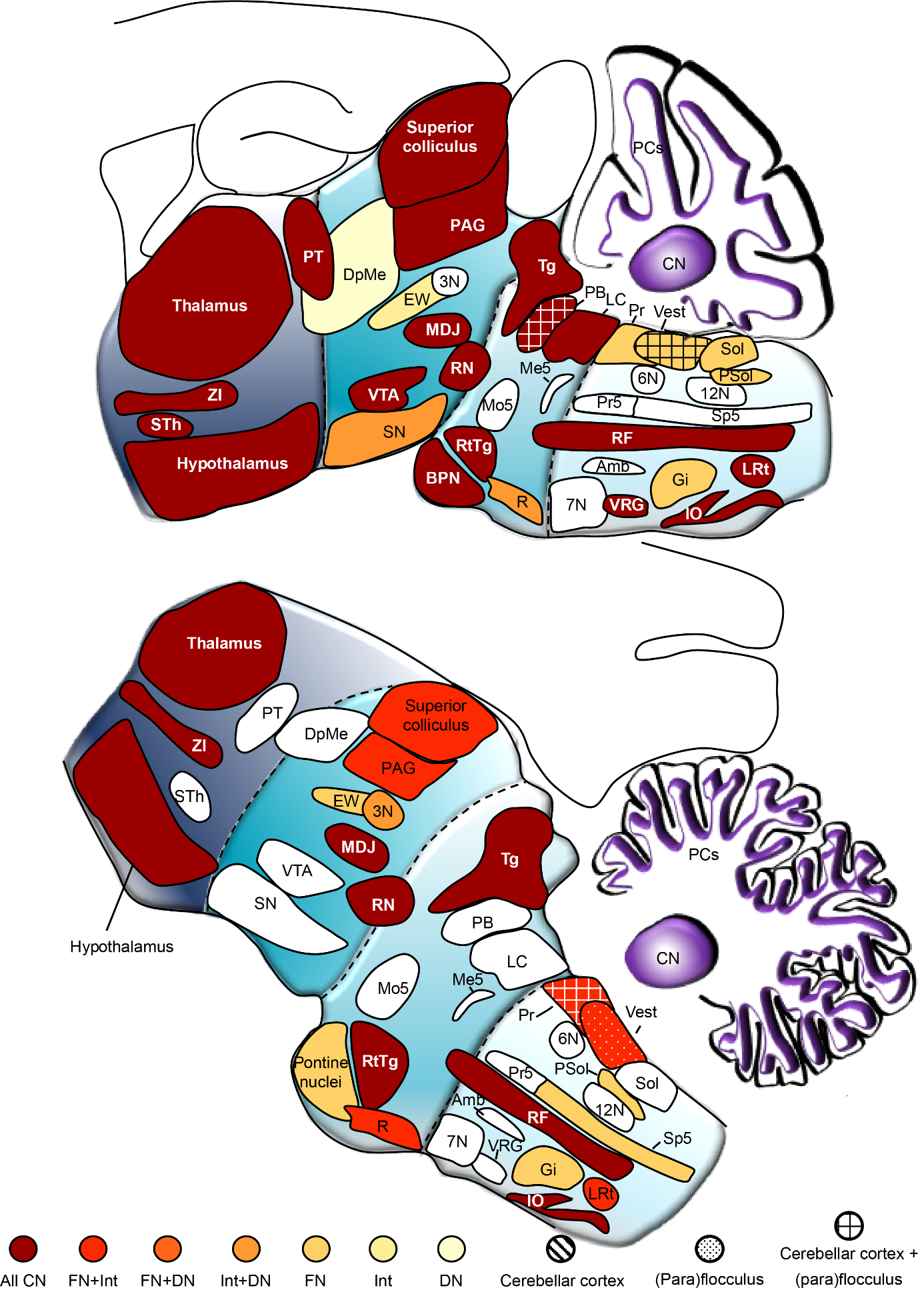


***Figure S5. Cerebellar projection pattern in rodents vs. non-human primates.***

*Same as figure S1, but for rodents (top), and non-human primates (bottom) using classical tracers.*
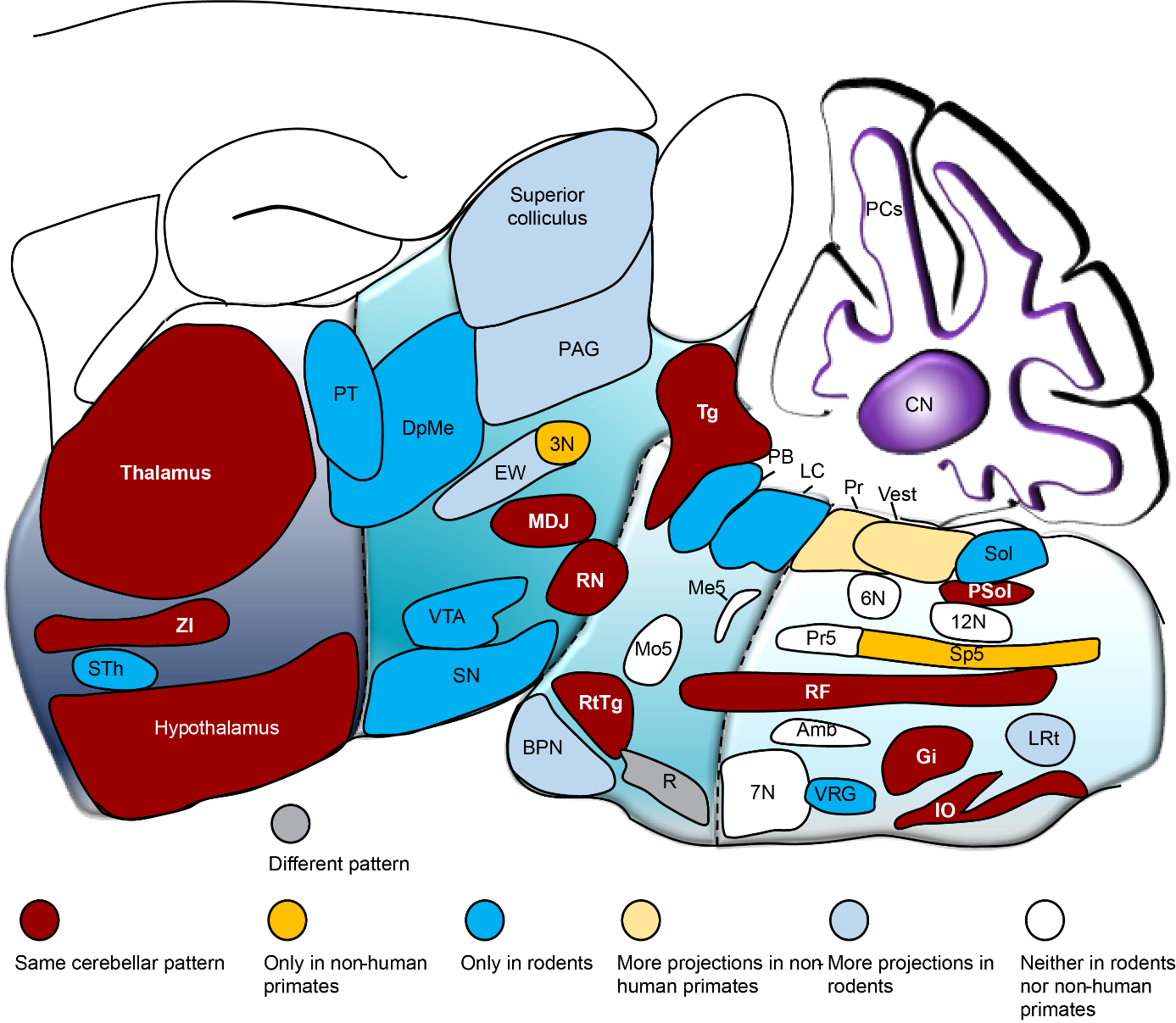


***Figure S6. Differences and similarities in rodents vs. non-human primates.***

*Same as figure S2, but for rodents and non-human primates using classical tracers.*

**Table S1 – Search queries**

| **Database searched** | **via** | **Years of coverage** | **References** | **After de-duplication** |
| --- | --- | --- | --- | --- |
| Embase | Embase.com | 1971 - Present | 5013 | 4949 |
| Medline ALL | Ovid | 1946 - Present | 4909 | 1435 |
| Web of Science Core Collection^1)^ | Web of Knowledge | 1975 - Present | 3221 | 934 |
| Cochrane Central Register of Controlled Trials | Wiley | 1992 - Present | 24 | 9 |
| **Total** |  |  | **13167** | **7327** |
| *Other sources: Google Scholar* | | | *200* | *77* |
| ***Total in .enlx file*** | | | ***13367*** | ***7404*** |
| **Search query for Embase**  ('efferent nerve'/exp OR 'neuronal tract tracer'/exp OR 'nerve tract'/de OR 'monosynaptic pathway'/de OR 'synaptic transmission'/de OR 'neurotransmission'/de OR (efferent* OR afferent* OR monosynap* OR neurotransmission* OR projection* OR ((neuro* OR nerve* OR neural* OR retrograde* OR anterograde* OR inject*) NEAR/3 (tracer* OR tracing)) OR ((neuro* OR nerve* OR neural* OR cerebellum* OR cerebellar* OR synap* OR fibre* OR fiber*) NEAR/3 (tract* OR pathway* OR circuit* OR connect* OR interconnect* OR transmiss*))):ab,ti,kw) **AND** ('cerebellum nucleus'/exp OR 'cerebellum'/de OR (cerebellum* OR cerebellar* OR ((dentat* OR fastigi* OR interpositus*) NEAR/3 (nucleus* OR nuclei))):ab,ti,kw) **AND** ('brain stem'/de OR 'cuneate nucleus'/de OR 'geniculate nucleus'/de OR 'gracile nucleus'/exp OR 'lateral reticular nucleus'/de OR 'locus ceruleus'/de OR 'magnocellular nucleus'/de OR 'median raphe nucleus'/de OR 'medulla oblongata'/exp OR 'mesencephalon'/exp OR 'parabrachial nucleus'/de OR 'respiration center'/de OR 'reticular formation'/exp OR 'trigeminal nucleus'/exp OR 'vagus nerve nucleus'/exp OR (brainstem* OR brain-stem* OR ((cuneat* OR geniculat* OR gracil* OR reticular* OR magnocellular* OR raphe* OR trigemin* OR ambiguus* OR hypoglossal* OR olivary* OR solitary-tract* OR vagus* OR vagal* OR vagi* OR interpeduncular* OR cuneiform* OR oculomotor* OR accessorius-nervi* OR westphal* OR tegment* OR trigemin* OR red OR rostral-interstitial* OR trochlear* OR parabrachial* OR raphe*) NEAR/3 (nucleus* OR nuclei* OR nuclear*)) OR ((oblongata* OR ventrolateral* OR ventromedial* OR brain* OR nucleus* OR pyramid* OR reticular*) NEAR/3 (medulla*)) OR ((aqueduct* OR aquaduct* OR aquaeduct* OR interventricular-duct* OR pedunc* OR crus OR crura OR trunc*) NEAR/3 (brain* OR cerebri* OR cerebral* OR sylvi*)) OR ((respirat*) NEAR/3 (center*)) OR ((reticular*) NEAR/3 (format* OR substan* OR system*)) OR ((ceruleus* OR caeruleus* OR coeruleus* OR coerulus*) NEAR/3 (locus* OR loci*)) OR area-postrema* OR mecencephal* OR midbrain* OR mid-brain* OR isthmic-organizer* OR substantia-nigra* OR tectum* OR colliculus* OR quadrigemina* OR posterior-commisure* OR tegmentum* OR periaqueductal-gray*):ab,ti,kw) NOT ([Conference Abstract]/lim) | | | | |
| **Search query for Medline**  (Neurons, Efferent/ OR Neuronal Tract-Tracers/ OR Neuroanatomical Tract-Tracing Techniques/ OR exp Neural Pathways/ OR exp Synaptic Transmission/ OR (efferent* OR afferent* OR monosynap* OR neurotransmission* OR projection* OR ((neuro* OR nerve* OR neural* OR retrograde* OR anterograde* OR inject*) ADJ3 (tracer* OR tracing)) OR ((neuro* OR nerve* OR neural* OR cerebellum* OR cerebellar* OR synap* OR fibre* OR fiber*) ADJ3 (tract* OR pathway* OR circuit* OR connect* OR interconnect* OR transmiss*))).ab,ti,kf.) AND (Cerebellar Nuclei/ OR Cerebellum/ OR (cerebellum* OR cerebellar* OR ((dentat* OR fastigi* OR interpositus*) ADJ3 (nucleus* OR nuclei))).ab,ti,kf.) AND (Brain Stem/ OR exp Mesencephalon/ OR exp Reticular Formation/ OR exp Trigeminal Nuclei/ OR (brainstem* OR brain-stem* OR ((cuneat* OR geniculat* OR gracil* OR reticular* OR magnocellular* OR raphe* OR trigemin* OR ambiguus* OR hypoglossal* OR olivary* OR solitary-tract* OR vagus* OR vagal* OR vagi* OR interpeduncular* OR cuneiform* OR oculomotor* OR accessorius-nervi* OR westphal* OR tegment* OR trigemin* OR red OR rostral-interstitial* OR trochlear* OR parabrachial* OR raphe*) ADJ3 (nucleus* OR nuclei* OR nuclear*)) OR ((oblongata* OR ventrolateral* OR ventromedial* OR brain* OR nucleus* OR pyramid* OR reticular*) ADJ3 (medulla*)) OR ((aqueduct* OR aquaduct* OR aquaeduct* OR interventricular-duct* OR pedunc* OR crus OR crura OR trunc*) ADJ3 (brain* OR cerebri* OR cerebral* OR sylvi*)) OR ((respirat*) ADJ3 (center*)) OR ((reticular*) ADJ3 (format* OR substan* OR system*)) OR ((ceruleus* OR caeruleus* OR coeruleus* OR coerulus*) ADJ3 (locus* OR loci*)) OR area-postrema* OR mecencephal* OR midbrain* OR mid-brain* OR isthmic-organizer* OR substantia-nigra* OR tectum* OR colliculus* OR quadrigemina* OR posterior-commisure* OR tegmentum* OR periaqueductal-gray*).ab,ti,kf.) NOT (news OR congres* OR abstract* OR book* OR chapter* OR dissertation abstract*).pt. | | | | |
| **Search query for Cochrane**  ((efferent* OR afferent* OR monosynap* OR neurotransmission* OR projection* OR ((neuro* OR nerve* OR neural* OR retrograde* OR anterograde* OR inject*) NEAR/3 (tracer* OR tracing)) OR ((neuro* OR nerve* OR neural* OR cerebellum* OR cerebellar* OR synap* OR fibre* OR fiber*) NEAR/3 (tract* OR pathway* OR circuit* OR connect* OR interconnect* OR transmiss*))):ab,ti,kw) **AND** ((cerebellum* OR cerebellar* OR ((dentat* OR fastigi* OR interpositus*) NEAR/3 (nucleus* OR nuclei))):ab,ti,kw) **AND** ((brainstem* OR (brain NEXT/1 stem*) OR ((cuneat* OR geniculat* OR gracil* OR reticular* OR magnocellular* OR raphe* OR trigemin* OR ambiguus* OR hypoglossal* OR olivary* OR (solitary NEXT/1 tract*) OR vagus* OR vagal* OR vagi* OR interpeduncular* OR cuneiform* OR oculomotor* OR (accessorius NEXT/1 nervi*) OR westphal* OR tegment* OR trigemin* OR red OR (rostral NEXT/1 interstitial*) OR trochlear* OR parabrachial* OR raphe*) NEAR/3 (nucleus* OR nuclei* OR nuclear*)) OR ((oblongata* OR ventrolateral* OR ventromedial* OR brain* OR nucleus* OR pyramid* OR reticular*) NEAR/3 (medulla*)) OR ((aqueduct* OR aquaduct* OR aquaeduct* OR (interventricular NEXT/1 duct*) OR pedunc* OR crus OR crura OR trunc*) NEAR/3 (brain* OR cerebri* OR cerebral* OR sylvi*)) OR ((respirat*) NEAR/3 (center*)) OR ((reticular*) NEAR/3 (format* OR substan* OR system*)) OR ((ceruleus* OR caeruleus* OR coeruleus* OR coerulus*) NEAR/3 (locus* OR loci*)) OR (area NEXT/1 postrema*) OR mecencephal* OR midbrain* OR (mid NEXT/1 brain*) OR (isthmic NEXT/1 organizer*) OR (substantia NEXT/1 nigra*) OR tectum* OR colliculus* OR quadrigemina* OR (posterior NEXT/1 commisure*) OR tegmentum* OR (periaqueductal NEXT/1 gray*)):ab,ti,kw) | | | | |
| **Search query for Web of Science**  TS=(((efferent* OR afferent* OR monosynap* OR neurotransmission* OR projection* OR ((neuro* OR nerve* OR neural* OR retrograde* OR anterograde* OR inject*) NEAR/2 (tracer* OR tracing)) OR ((neuro* OR nerve* OR neural* OR cerebellum* OR cerebellar* OR synap* OR fibre* OR fiber*) NEAR/2 (tract* OR pathway* OR circuit* OR connect* OR interconnect* OR transmiss*)))) AND ((cerebellum* OR cerebellar* OR ((dentat* OR fastigi* OR interpositus*) NEAR/2 (nucleus* OR nuclei)))) AND ((brainstem* OR brain-stem* OR ((cuneat* OR geniculat* OR gracil* OR reticular* OR magnocellular* OR raphe* OR trigemin* OR ambiguus* OR hypoglossal* OR olivary* OR solitary-tract* OR vagus* OR vagal* OR vagi* OR interpeduncular* OR cuneiform* OR oculomotor* OR accessorius-nervi* OR westphal* OR tegment* OR trigemin* OR red OR rostral-interstitial* OR trochlear* OR parabrachial* OR raphe*) NEAR/2 (nucleus* OR nuclei* OR nuclear*)) OR ((oblongata* OR ventrolateral* OR ventromedial* OR brain* OR nucleus* OR pyramid* OR reticular*) NEAR/2 (medulla*)) OR ((aqueduct* OR aquaduct* OR aquaeduct* OR interventricular-duct* OR pedunc* OR crus OR crura OR trunc*) NEAR/2 (brain* OR cerebri* OR cerebral* OR sylvi*)) OR ((respirat*) NEAR/2 (center*)) OR ((reticular*) NEAR/2 (format* OR substan* OR system*)) OR ((ceruleus* OR caeruleus* OR coeruleus* OR coerulus*) NEAR/2 (locus* OR loci*)) OR area-postrema* OR mecencephal* OR midbrain* OR mid-brain* OR isthmic-organizer* OR substantia-nigra* OR tectum* OR colliculus* OR quadrigemina* OR posterior-commisure* OR tegmentum* OR periaqueductal-gray*))) AND DT=(Article OR Review OR Letter OR Early Access) | | | | |
| **Search query for Google Scholar**  efferent\|efferents\|afferent\|afferents cerebellum\|cerebellar\|"dentate\|fastigial\|interpositus nucleus\|nuclei" brainstem\|"brain*stem" | | | | |

^1)^ Science Citation Index Expanded (1975-present); Social Sciences Citation Index (1975-present); Arts & Humanities Citation Index (1975-present); Conference Proceedings Citation Index- Science (1990-present); Conference Proceedings Citation Index- Social Science & Humanities (1990-present) ; Emerging Sources Citation Index (2015-present)

**Table S2 – Monosynaptic connections**

| **Target area**  Title: The table represents all brainstem and diencephalic structures target indicating for each paper what is the cerebellar sources, which tracer has been used, and if it was antero- or retro-grade, and which animal has been used. | **Cerebellar cortex** | **FN** | **DLP** | **Int** | **DLH** | **DN** | **A/R** | **Animal** | **Tracer** | **References** |
| --- | --- | --- | --- | --- | --- | --- | --- | --- | --- | --- |
| **Medulla oblongata** | No | Yes | No | Yes | Yes | Yes | R | Rat | NY, BB, GB | [44] |
|  | No | Yes | No | Yes | Yes | Yes | R | Rat | FB | [45] |
|  | No | No | No | No | No | Yes | A/R | Rat | Ctb, BDA | [46] |
|  | | | | | | | | | | |
| **Reticular formation** | No | Yes | No | No | No | Yes | R | Rat | HRP | [201] |
|  | N/A | Yes | N/A | N/A | N/A | N/A | A | Cat | AR | [74] |
|  | No | Yes | No | No | No | No | R | Cat | AR | [83] |
|  | N/A | Yes | N/A | Yes | N/A | Yes | A | Monkey^a^ | AR | [65] |
|  | N/A | Yes | N/A | N/A | N/A | N/A | A | Dog | AR | [49] |
|  | N/A | N/A | N/A | Yes | N/A | Yes | A | Cat | AR | [187] |
|  | N/A | Yes | Yes | Yes | Yes | Yes | A/R | Rat | Ctb, BDA | [484] |
|  | N/A | Yes | No | N/A | N/A | N/A | A | Mouse | AAV | [33] |
|  | N/A | No | N/A | No | N/A | Yes | A | Mouse | AAV | [34] |
|  | N/A | N/A | N/A | Yes | N/A | N/A | A | Mouse | AAV | [32] |
|  | | | | | | | | | | |
| **Mesencephalic reticular formation** | No | Yes | Yes | Yes | Yes | Yes | R | Rat | EB, TB | [44] |
|  | N/A | Yes | N/A | No | N/A | No | A | Monkey^a^ | AR | [65] |
|  | No | Yes | No | Yes | Yes | Yes | R | Rat | HRP | [206] |
|  | N/A | Yes | Yes | N/A | N/A | N/A | A | Mouse | AAV | [33] |
|  | N/A | N/A | N/A | Yes | N/A | N/A | A | Mouse | AAV | [32] |
|  | | | | | | | | | | |
| **Nucleus of the posterior commissure** | N/A | Yes | N/A | N/A | N/A | N/A | A | Monkey | AR | [54] |
|  | N/A | No | N/A | No^2^ | N/A | Yes | A | Cat | AR | [48] |
|  | N/A | No | N/A | No | N/A | Yes | A | Cat | AR | [207] |
|  | N/A | Yes | N/A | N/A | N/A | N/A | A | Dog | AR | [49] |
|  | N/A | Yes | N/A | N/A | N/A | N/A | A | Dog | AR | [208] |
|  | N/A | N/A | N/A | Yes | N/A | N/A | A | Monkey^b^ | HRP | [67] |
|  | N/A | No | N/A | No | N/A | Yes | A | Rat | WGA-HRP | [209] |
|  | | | | | | | | | | |
| **Pontine reticular formation** | No | Yes | Yes | Yes | Yes | Yes | R | Rat | HRP | [176] |
|  | N/A | Yes | N/A | Yes | N/A | Yes | A | Monkey^a^ | AR | [65] |
|  | N/A | Yes | Yes | Yes | Yes | Yes | R | Rat | HRP | [177] |
|  | N/A | N/A | N/A | Yes^1^ | N/A | N/A | A | Cat | AR | [55] |
|  | No | Yes | No | Yes | No | Yes | R | Rat | TB, DY | [173] |
|  | No | Yes | No | No | No | Yes | R | Cat | HRP | [174] |
|  | N/A | Yes | Yes | Yes | Yes | Yes | A | Monkey^c^ | Biocytin | [175] |
|  | N/A | No | Yes | N/A | N/A | N/A | A | Mouse | AAV | [33] |
|  | | | | | | | | | | |
| **PMf** | N/A | Yes | N/A | N/A | N/A | N/A | A | Cat | AR | [47] |
|  | N/A | Yes | N/A | N/A | N/A | N/A | A | Monkey | AR | [54] |
|  | N/A | Yes | N/A | N/A | N/A | N/A | A | Dog | AR | [49] |
|  | No | Yes | Yes | Yes | Yes | Yes | R | Cat | HRP | [178] |
|  | No | Yes | No | No | No | No | R | Monkey^d^ | WGA | [179] |
| **PMf** | No | Yes | No | No | No | No | R | Monkey^e^ | HRP | [79] |
|  | No | Yes | No | No | No | No | R | Monkey^f^ | FB, DY | [180] |
|  | N/A | Yes | N/A | N/A | N/A | N/A | A | Cat | PHA-L | [51] |
|  | N/A | Yes | N/A | N/A | N/A | N/A | A | Cat | DB | [57] |
|  | N/A | Yes | No | N/A | N/A | N/A | A | Mouse | AAV | [33] |
|  | | | | | | | | | | |
| **Nucleus reticularis tegmenti pontis** | No | No | No | Yes^1^ | No | No | R | Cat | HRP | [105] |
|  | N/A | Yes | N/A | N/A | N/A | N/A | A | Rat | AR | [186] |
|  | N/A | Yes | N/A | Yes | N/A | Yes | A | Monkey^a^ | AR | [65] |
|  | N/A | N/A | N/A | Yes | N/A | Yes | A | Cat | AR | [187] |
|  | N/A | N/A | N/A | Yes | N/A | Yes | A | Rat | AR | [188] |
|  | N/A | N/A | N/A | Yes^1^ | N/A | N/A | A | Cat | AR | [55] |
|  | No | Yes | No | Yes^1^ | Yes | Yes | R | Rat | HRP | [189] |
|  | No | No | No | No | No | Yes | R | Monkey^e^ | HRP | [79] |
|  | N/A | Yes | N/A | N/A | N/A | N/A | A | Cat | PHA-L | [51] |
|  | N/A | N/A | N/A | N/A | N/A | Yes | A | Rat | BDA | [117] |
|  | N/A | N/A | N/A | Yes | N/A | Yes | A | Cat | WGA-HRP | [190] |
|  | N/A | N/A | N/A | N/A | N/A | Yes | A | Monkey^g^ | AR | [191] |
|  | N/A | Yes | N/A | N/A | N/A | N/A | A | Rat | WGA-HRP | [56] |
|  | No | Yes | No | Yes | Yes | Yes | R | Rat | Ctb, GL | [110] |
|  | N/A | Yes | N/A | N/A | N/A | N/A | A | Cat | DB | [57] |
|  | No | Yes | Yes | Yes | Yes | Yes | A/R | Rat | BDA, FG | [64] |
|  | N/A | Yes | Yes | N/A | N/A | N/A | A | Mouse | AAV | [33] |
|  | N/A | N/A | N/A | Yes | N/A | N/A | A | Mouse | AAV | [32] |
|  | | | | | | | | | | |
| **Medullary reticular formation** | N/A | Yes | N/A | N/A | N/A | N/A | A | Cat | AR | [47] |
|  | No | Yes | No | Yes | No | No | R | Cat | NY | [485] |
|  | No | Yes | No | Yes | No | Yes | R | Rat | NY | [44] |
|  | No | Yes | No | No | No | No | A | Cat | AR | [48] |
|  | N/A | Yes | N/A | N/A | N/A | N/A | A | Dog | AR | [49] |
|  | No | Yes | No | No | No | No | R | Cat | WGA-HRP | [50] |
|  | N/A | Yes | N/A | N/A | N/A | N/A | A | Cat | PHA-L | [51] |
|  | No | Yes | No | No | No | No | A/R | Mouse | Dextran,  Tracer Dye | [52] |
|  | No | Yes | No | Yes | No | No | A/R | Mouse | BDA, FG | [53] |
|  | N/A | Yes | No | N/A | N/A | N/A | A | Mouse | AAV | [33] |
|  | N/A | Yes | N/A | Yes | N/A | No | A | Mouse | AAV | [34] |
|  | N/A | N/A | N/A | Yes | N/A | N/A | A | Mouse | AAV | [32] |
|  | | | | | | | | | | |
| **Gi** | N/A | Yes | N/A | N/A | N/A | N/A | A | Cat | AR | [47] |
|  | N/A | Yes | N/A | N/A | N/A | N/A | A | Monkey | AR | [54] |
|  | N/A | Yes | N/A | N/A | N/A | N/A | A | Dog | AR | [49] |
|  | N/A | N/A | N/A | Yes | N/A | N/A | A | Cat | AR | [55] |
|  | N/A | Yes | N/A | N/A | N/A | N/A | A | Cat | PHA-L | [51] |
|  | No | Yes | No | No | No | No | R | Rat | HRP | [58] |
| **Gigantocellular nucleus** | N/A | Yes | N/A | N/A | N/A | N/A | A | Rat | WGA-HRP | [56] |
|  | N/A | Yes | N/A | N/A | N/A | N/A | A | Mouse | BDA | [53] |
|  | N/A | Yes | N/A | N/A | N/A | N/A | A | Cat | DB | [57] |
|  | No | Yes | Yes | No | No | No | A/R | Mouse | AAV, FB, or retrobeads. | [33] |
|  | N/A | Yes | N/A | Yes | N/A | No | A | Mouse | AAV | [34] |
|  | N/A | N/A | N/A | Yes | N/A | N/A | A | Mouse | AAV | [32] |
|  | | | | | | | | | | |
| **PGi** | N/A | Yes | N/A | N/A | N/A | N/A | R | Mouse | BDA | [53] |
|  | No | Yes | No | No | No | No | A/R | Mouse | AAV | [33] |
|  | N/A | Yes | N/A | Yes | N/A | Yes | A | Mouse | AAV | [34] |
|  | N/A | N/A | N/A | Yes | N/A | N/A | A | Mouse | AAV | [32] |
|  | | | | | | | | | | |
| **Lateral reticular nucleus** | N/A | Yes | N/A | N/A | N/A | N/A | A | Cat | AR | [47] |
|  | N/A | Yes | N/A | N/A | N/A | N/A | A | Monkey | AR | [54] |
|  | No | Yes | Yes | Yes | Yes | Yes | A/R | Cat | HRP, AR | [74] |
|  | N/A | N/A | N/A | Yes | N/A | N/A | A | Cat | AR | [55] |
|  | N/A | Yes | N/A | Yes | N/A | Yes | R | Cat | WGA-HRP | [75] |
|  | N/A | N/A | N/A | Yes | N/A | N/A | A | Monkey^b^ | HRP | [67] |
|  | N/A | Yes | N/A | N/A | N/A | N/A | A | Rat | WGA-HRP | [76] |
|  | N/A | Yes | N/A | N/A | N/A | N/A | A | Cat | AR | [51] |
|  | No | No | No | No | No | Yes | A/R | Rat | Ctb, BDA | [46] |
|  | N/A | N/A | N/A | Yes | N/A | N/A | A | Mouse | AAV | [77] |
|  | N/A | Yes | N/A | Yes | N/A | Yes | A | Mouse | AAV | [34] |
|  | N/A | N/A | N/A | Yes | N/A | N/A | A | Mouse | AAV | [32] |
|  | | | | | | | | | | |
| **Parvocellular reticular nuclei** | N/A | Yes | N/A | N/A | N/A | N/A | A | Cat | AR | [47] |
|  | N/A | Yes | Yes | N/A | N/A | N/A | A | Mouse | AAV | [33] |
|  | N/A | Yes | N/A | Yes | N/A | Yes | A | Mouse | AAV | [34] |
|  | N/A | N/A | N/A | Yes | N/A | N/A | A | Mouse | AAV | [32] |
|  | | | | | | | | | | |
|  | N/A | Yes | N/A | N/A | N/A | N/A | A | Monkey | AR | [54] |
| **mRtn** | N/A | Yes | N/A | N/A | N/A | N/A | A | Dog | AR | [49] |
|  | N/A | Yes | N/A | N/A | N/A | N/A | A | Cat | PHA-L | [51] |
|  | N/A | Yes | N/A | No^1^ | N/A | Yes | A | Mouse | AAV | [34] |
|  | N/A | N/A | N/A | Yes | N/A | N/A | A | Mouse | AAV | [32] |
|  | | | | | | | | | | |
| **Tg** | No | No | No | Yes | No | Yes | R | Rat | HRP | [84] |
|  | No | Yes | No | No | No | No | A | Cat | AR | [48] |
|  | No | No | No | No | No | Yes | R | Cat | AR | [83] |
|  | No | No | N/A | No | N/A | Yes | A | Rat | AR | [172] |
|  | N/A | N/A | N/A | Yes | N/A | Yes | A | Cat | AR | [187] |
|  | No | Yes | Yes | Yes | Yes | Yes | R | Cat | HRP | [80] |
| **Tegmentum** | No | No | No | Yes | No | Yes | R | Rat | HRP | [85] |
|  | No | Yes | No | No | No | No | A/R | Bat | WGA-HRP | [82] |
|  | N/A | Yes | Yes | Yes | Yes | Yes | R | Rat | FG | [81] |
|  | N/A | Yes | No | N/A | N/A | N/A | A | Mouse | AAV | [33] |
|  | N/A | N/A | N/A | Yes | N/A | N/A | A | Mouse | AAV | [32] |
|  | | | | | | | | | | |
| **Raphe nuclei** | No | Yes | No | No | No | No | A | Monkey^a^ | AR | [65] |
|  | No | Yes | No | No | No | No | R | Cat | HRP | [66] |
|  | N/A | N/A | N/A | Yes | N/A | N/A | A | Cat | AR | [55] |
|  | No | No | No | Yes^2^ | No | Yes | R | Rat | WGA-HRP | [68] |
|  | N/A | N/A | N/A | Yes | N/A | N/A | A | Monkey^b^ | HRP | [67] |
|  | No | Yes | Yes | Yes | Yes | Yes | A/R | Rat | BDA, FG | [64] |
|  | N/A | Yes | N/A | Yes | N/A | Yes | A | Mouse | AAV | [34] |
|  | N/A | N/A | N/A | Yes | N/A | N/A | A | Mouse | AAV | [32] |
|  | | | | | | | | | | |
| **Inferior olive** | N/A | N/A | N/A | Yes  MAO, DAO | N/A | Yes  PO, vlp | A | Cat | AR | [108] |
|  | N/A | Yes  MAO. | N/A | Yes  MAO, PO, DAO, dmcl | N/A | Yes  MAO, PO | A | Opossum | AR | [94] |
|  | N/A | N/A | N/A | Yes  MAO, DAO | N/A | Yes  PO. | A | Cat | AR | [107] |
|  | N/A | Yes  MAO, DAO | N/A | N/A | N/A | N/A | A | Monkey | AR | [54] |
|  | No | No | No | Yes^2^ | No | Yes  vlp | R | Cat | HRP | [114] |
|  | No | No | No | Yes | No | No | R | Cat | HRP | [105] |
|  | No | No | No | Yes  DAO | No | Yes  PO | A | Monkey^d^ | AR | [113] |
| **IO** | No | Yes  MAO | No | Yes  DAO | No | Yes  PO, DAO | R | Cat | HRP | [90] |
|  | No | Yes  MAO, vlp, DAO | No | Yes  MAO, PO, dmcl | No | Yes  PO, dmcl | A | Rat | AR | [97] |
|  | No | No | No | Yes | Yes | Yes | R | Rat | NY, BB, GB | [44] |
|  | No | No | No | No | No | Yes | R | Rat | HRP | [116] |
|  | No | No | No | Yes  MAO, DAO, Kooy | No | Yes  PO | A | Monkey^a^ | AR | [65] |
|  | N/A | Yes  MAO, Kooy, β | N/A | N/A | N/A | N/A | A | Cat | WGA-HRP | [101] |
|  | N/A | N/A | N/A | N/A | N/A | Yes  PO, vlp | A | Cat | WGA-HRP | [119] |
| **Inferior olive** | N/A | N/A | N/A | Yes^1^ | N/A | N/A | A | Cat | AR | [55] |
|  | N/A | N/A | N/A | Yes  DAO, β | N/A | N/A | A | Cat | WGA-HRP | [112] |
|  | N/A | N/A | N/A | N/A | N/A | Yes  PO | A | Rat | AR | [98] |
|  | N/A | N/A | N/A | Yes  MAO, PO | N/A | Yes  MAO, PO | A | Cat | WGA-HRP | [109] |
|  | N/A | N/A | N/A | N/A | N/A | Yes | A | Rat | WGA-HRP | [99] |
|  | No | Yes  MAO | No | Yes | No | No | A | Monkey^f^ | WGA-HRP | [100] |
|  | No | Yes | Yes | Yes | Yes | yes | R | Rat | WGA-HRP | [92] |
| **Inferior olive** | No | Yes | No | Yes | No | Yes | A | Cat | WGA-HRP, AR | [96] |
|  | N/A | N/A | N/A | Yes  MAO, DAO. | N/A | N/A | A | Monkey^b^ | HRP | [67] |
|  | No | Yes  MAO, β, dmcl | Yes  MAO | Yes  MAO, DAO | Yes | Yes  PO, vlp | A | Rat | PHA-L | [102] |
|  | No | Yes | No | Yes  DAO. | Yes  MAO. | Yes  PO. | R | Rat | WGA-HRP | [91] |
|  | No | No | No | No | No | Yes  MAO, PO, vlp, Kooy | A/R | Rabbit | HRP, WGA-HRP | [120] |
|  | No | Yes | Yes | Yes | Yes | Yes | R | Rat | WGA-BSA-gold | [95] |
|  | No | No | No | No | No | Yes | R | Rabbit | Ctb, DY | [118] |
| **Inferior olive** | N/A | N/A | N/A | N/A | Yes  T-area* | N/A | A | Rat | PHA-L | [115] |
|  | N/A | N/A | N/A | N/A | N/A | Yes | A | Rat | BDA | [117] |
|  | No | No | No | Yes | No | Yes | A | Hedgehog | WGA-HRP, BDA | [104] |
|  | No | No | No | Yes | Yes | Yes | R | Rat | GL | [121] |
|  | No | No | No | Yes | No | Yes | R | Mouse | Fluorescent microspheres | [106] |
|  | No | Yes  MAO | No | No | No | No | R | Rat | Gold-HRP | [103] |
|  | No | No | No | Yes^1^  PO | No | Yes  PO, vlp, DAO | A | Cat | WGA-HRP | [111] |
| **Inferior olive** | N/A | Yes  MAO. | N/A | N/A | N/A | N/A | A | Rat | WGA-HRP | [56] |
|  | No | Yes | Yes | Yes | Yes | Yes | A | Mouse | H129ΔTK-TT virus | [93] |
|  | No | No | No | Yes | No | Yes | R | Rat | Ctb, GL | [110] |
|  | N/A | N/A | N/A | Yes^1^  DAO | N/A | N/A | A | Mouse | AAV | [77] |
|  | N/A | Yes  MAO, β | Yes  MAO | N/A | N/A | N/A | A | Mouse | AAV | [33] |
|  | N/A | Yes | N/A | N/A | N/A | Yes | A | Mouse | AAV | [34] |
|  | N/A | N/A | N/A | Yes | N/A | N/A | A | Mouse | AAV | [32] |
|  | | | | | | | | | | |
| **Nucleus of the solitary tract** | N/A | Yes | N/A | N/A | N/A | N/A | A | Cat | AR | [47] |
|  | No | Yes | No | No | No | No | R | Rat | HRP | [129] |
|  | N/A | Yes | N/A | N/A | N/A | N/A | A | Dog | AR | [49] |
|  | N/A | N/A | N/A | Yes^1^ | N/A | N/A | A | Cat | AR | [55] |
|  | No | Yes | No | No | No | No | R | Cat | HRP | [128] |
|  | No | Yes | No | No | No | No | R | Rat | Rhodamine latex microbeads | [130] |
|  | N/A | N/A | N/A | Yes | N/A | N/A | A | Mouse | AAV | [32] |
|  | | | | | | | | | | |
| **Parasolitary nucleus** | N/A | Yes | N/A | N/A | N/A | N/A | A | Monkey | AR | [54] |
|  | N/A | Yes | N/A | N/A | N/A | N/A | A | Dog | AR | [49] |
|  | N/A | Yes | N/A | N/A | N/A | N/A | A | Cat | PHA-L | [51] |
|  | N/A | Yes | N/A | N/A | N/A | N/A | A | Rat | WGA-HRP | [56] |
|  | N/A | Yes | N/A | Yes | N/A | Yes | A | Mouse | AAV | [34] |
|  | | | | | | | | | | |
| **5N** | N/A | Yes | N/A | N/A | N/A | N/A | A | Monkey | AR | [54] |
|  | N/A | Yes | N/A | Yes | N/A | Yes | A | Mouse | AAV | [34] |
|  | N/A | N/A | N/A | Yes | N/A | N/A | A | Mouse | AAV | [32] |
|  | | | | | | | | | | |
| **Vestibular nuclei** | N/A | Yes  MVN, LVN, SpVN | N/A | N/A | N/A | N/A | A | Cat | AR | [47] |
|  | N/A | Yes  LVN, SpVN, MVN, SVN | N/A | N/A | N/A | N/A | A | Monkey | AR | [54] |
|  | Yes  MVN | Yes  MVN | Yes  MVN | Yes  MVN | Yes  MVN | Yes  MVN | R | Rabbt | HRP | [154] |
|  | N/A | Yes  LVN. | N/A | N/A | N/A | N/A | A | Cat | AR | [48] |
|  | Yes  LVN | Yes  LVN | N/A | Yes  LVN | N/A | Yes  LVN | R | Cat | HRP | [40] |
|  | Yes  MVN, LVN, SVN | No | No | No | No | No | R | Cat | HRP | [137] |
|  | N/A | Yes  LVN, SpVN | No | No | No | No | A | Monkey^a^ | AR | [65] |
|  | Yes  MVN, SpVN, LVN | Yes | No | No | No | No | R | Cat | HRP | [138] |
|  | Yes  LVN. | Yes | No | No | No | No | R | Monkey^c^ | HRP | [138] |
|  | N/A | Yes  LVN, SpVN, MVN | N/A | N/A | N/A | N/A | A | Dog | AR | [49] |
|  | Yes  SVN | No | No | No | No | No | A/R | Cat | HRP, WGA-HRP, AR | [139] |
|  | Yes  SVN, MVN, LVN | N/A | N/A | N/A | N/A | N/A | A | Rhesus macaques | AR | [140] |
|  | N/A | N/A | N/A | Yes  SVN, LVN | N/A | N/A | A | Cat | AR | [55] |
|  | Yes | N/A | N/A | N/A | N/A | N/A | A | Rat | WGA-HRP | [150] |
|  | Yes  SpVN, MVN, SVN | No | No | No | No | No | R | Cat | HRP | [141] |
|  | N/A | N/A | N/A | Yes  MVN | N/A | N/A | A | Monkey | HRP | [67] |
|  | Yes | N/A | N/A | N/A | N/A | N/A | A | Rat | HRP | [142] |
|  | Yes  MVN, SpVN, LVN, SVN | N/A | N/A | N/A | N/A | N/A | A | Rabbit | Biocytin | [143] |
| **Vestibular nuclei** | Yes  SVN, MVN | N/A | N/A | N/A | N/A | N/A | A | Rabbit | WGA-HRP, biocytin | [151] |
|  | N/A | Yes  SVN, LVN, SpVN, MVN | N/A | N/A | N/A | N/A | A | Cat | PHA-L | [51] |
|  | Yes  SVN, MVN | N/A | N/A | N/A | N/A | N/A | A | Rabbit | BDA | [144] |
|  | No | Yes | No | No | No | No | R | Rat | Gold-HRP, BDA | [103] |
|  | N/A | Yes  SVN, IVN, LVN, MVN | N/A | N/A | N/A | N/A | A | Rat | WGA-HRP | [56] |
|  | Yes  LVN, IVN, SVN | N/A | N/A | N/A | N/A | N/A | A | Rat | BDA | [153] |
|  | N/A | Yes  LVN, IVN | N/A | N/A | N/A | N/A | A/R | Mouse | Dextran, dye | [52] |
|  | No | Yes | Yes | Yes | Yes | Yes | A | Mouse | H129ΔTK-TT virus | [93] |
|  | Yes | N/A | N/A | N/A | N/A | N/A | A | Rat | Lentivirus vector | [145] |
|  | Yes  MVN | No | No | No | No | No | R | Mouse | Intracellular biocytin | [146] |
|  | Yes | No | No | No | No | No | R | Rat | FG | [147] |
|  | Yes  SVN, MVN | N/A | N/A | N/A | N/A | N/A | A | Mouse | LV | [152] |
|  | N/A  SpVN, MVN | N/A | N/A | N/A | N/A | N/A | A/R | Rat | BDA, FG | [64] |
|  | Yes  MVN, SVN, LVN, SpVN | N/A | N/A | N/A | N/A | N/A | A | Mouse | AAV | [41] |
|  | N/A | Yes  LVN, SpVN | N/A | N/A | N/A | N/A | A | Mouse | AAV | [33] |
|  | N/A | Yes  SpVN, SVN, MVN, LVN | N/A | Yes  SpVN, SVN, MVN, LVN | N/A | Yes  SpVN, SVN, LVN | A | Mouse | AAV | [34] |
|  | N/A | N/A | N/A | Yes  LVN, MVN | N/A | N/A | A | Mouse | AAV | [32] |
|  | Yes | No | No | No | No | No | A | Mouse | Herpes simplex virus | [148] |
|  | Yes | Yes | No | Yes | No | No | R | Mouse | RV | [149] |
|  | | | | | | | | | | |
| **Hypoglossal nucleus** | Yes | Yes | Yes | Yes | Yes | Yes | R | Mouse | RV | [156] |
|  | N/A | N/A | N/A | Yes | N/A | N/A | A | Mouse | AAV | [32] |
|  | | | | | | | | | | |
| **Pr** | N/A | Yes | N/A | N/A | N/A | N/A | A | Cat | AR | [47] |
|  | N/A | Yes | N/A | Yes | N/A | yes | A | Mouse | AAV | [34] |
|  | | | | | | | | | | |
| **Prepositus hypoglossi nucleus** | N/A | Yes | N/A | N/A | N/A | N/A | A | Monkey | AR | [54] |
|  | No | Yes | No | No | No | No | A | Monkey^a^ | AR | [65] |
|  | Yes | Yes | No | No | No | No | R | Cat | HRP | [164] |
|  | N/A | N/A | N/A | Yes | N/A | N/A | A | Cat | AR | [55] |
|  | Yes | Yes | No | No | No | No | R | Monkey^c^ | WGA-HRP | [165] |
|  | N/A | N/A | N/A | Yes | N/A | N/A | A | Monkey^b^ | HRP | [67] |
|  | N/A | Yes | N/A | N/A | N/A | N/A | A | Rat | WGA-HRP | [56] |
|  | N/A | Yes | N/A | N/A | N/A | N/A | A | Mouse | AAV | [33] |
|  | N/A | N/A | N/A | Yes | N/A | N/A | A | Mouse | AAV | [32] |
|  | | | | | | | | | | |
| **Amb** | N/A | Yes | N/A | N/A | N/A | N/A | A | Cat | AR | [47] |
|  | | | | | | | | | | |
| **VRC** | N/A | Yes | Yes | Yes | Yes | Yes | R | Rat | FB | [167] |
|  | | | | | | | | | | |
| **Locus Coeruleus** | N/A | Yes | N/A | N/A | N/A | N/A | A | Cat | AR | [47] |
|  | No | Yes | No | No | No | No | R | Rat | HRP | [201] |
|  | No | Yes | Yes | Yes | Yes | Yes | R | Rat | HRP | [176] |
|  | No | Yes | No | No | No | No | R | Rat | HRP | [200] |
|  | N/A | N/A | N/A | Yes^1^ | N/A | N/A | A | Cat | AR | [55] |
|  | N/A | Yes | N/A | N/A | N/A | N/A | A | Rat | WGA-HRP | [56] |
|  | Yes | Yes | Yes | Yes | Yes | Yes | A/R | Mouse | CAV, RV | [36] |
|  | N/A | Yes | N/A | N/A | N/A | N/A | A | Mouse | AAV | [33] |
|  | N/A | Yes | N/A | Yes | N/A | Yes | A | Mouse | AAV | [34] |
|  | | | | | | | | | | |
| **Pontine nuclei** | N/A | Yes | N/A | N/A | N/A | N/A | A | Cat | AR | [47] |
|  | N/A | Yes | N/A | N/A | N/A | N/A | A | Monkey | AR | [54] |
|  | No | No | No | Yes^2^ | No | No | R | Cat | HRP | [105] |
|  | No | Yes | Yes | Yes | Yes | Yes | R | Rat | HRP | [196] |
|  | N/A | Yes | N/A | N/A | N/A | N/A | A | Rat | AR | [186] |
|  | N/A | Yes | No | No | No | No | A | Monkey^a^ | AR | [65] |
|  | No | Yes | Yes | Yes | Yes | Yes | R | Rabbit | HRP | [195] |
|  | N/A | Yes | N/A | Yes | N/A | Yes | A | Rat | AR | [172] |
|  | N/A | N/A | N/A | Yes | N/A | Yes | A | Rat | AR | [188] |
|  | No | Yes | Yes | Yes | Yes | Yes | R | Rat | NY | [92] |
|  | No | Yes | Yes | Yes | Yes | Yes | A | Cat | WGA-HRP | [194] |
|  | N/A | N/A | N/A | N/A | N/A | Yes | A | Rat | BDA | [117] |
|  | No | Yes | No | Yes | No | Yes | R | Rat | GL | [110] |
|  | N/A | No | Yes | N/A | N/A | N/A | A | Mouse | AAV | [33] |
|  | | | | | | | | | | |
| **Parabrachial complex** | No | Yes | No | No | No | Yes | R | Rat | HRP | [201] |
|  | N/A | Yes | N/A | N/A | N/A | N/A | A | Dog | AR | [49] |
|  | Yes | Yes | N/A | N/A | N/A | N/A | A | Rabbit | WGA-HRP or Ctb-HRP | [204] |
|  | Yes | No | No | No | No | No | A/R | Rabbit | BDA, HRP | [144] |
|  | Yes | N/A | N/A | N/A | N/A | N/A | A | Rat | BDA | [153] |
|  | No | Yes | Yes | Yes | Yes | Yes | A/R | Rat | BDA, FG | [64] |
|  | Yes | Yes | No | Yes | No | No | A/R | Mouse | AAV, FB, rAAV | [41] |
|  | N/A | Yes | N/A | N/A | N/A | N/A | A | Mouse | AAV | [33] |
|  | N/A | Yes | N/A | Yes | N/A | Yes | A | Mouse | AAV | [34] |
|  | N/A | N/A | N/A | Yes | N/A | N/A | A | Mouse | AAV | [32] |
|  | | | | | | | | | | |
| **6N** | N/A | Yes | N/A | N/A | N/A | N/A | A | Cat | PHA-L | [51] |
|  | | | | | | | | | | |
| **7N** | N/A | Yes | N/A | N/A | N/A | N/A | A | Cat | PHA-L | [51] |
|  | N/A | Yes | Yes | N/A | N/A | N/A | A | Mouse | AAV | [33] |
|  | | | | | | | | | | |
| **Red nucleus** | N/A | Yes | N/A | N/A | N/A | N/A | A | Monkey | AR | [54] |
|  | No | No | No | Yes | Yes | Yes | R | Rat | HRP | [226] |
|  | No | No | No | Yes | No | No | R | Cat | HRP | [105] |
|  | N/A | Yes | Yes | Yes | Yes | Yes | A | Cat | AR | [227] |
|  | No | No | No | No | No | Yes | R | Rat | HRP | [228] |
|  | N/A | N/A | N/A | Yes | N/A | N/A | A | Rat | AR | [229] |
|  | N/A | Yes | N/A | Yes | N/A | Yes | A | Monkey^d^ | AR | [230] |
| **Red nucleus** | No | No | No | No | No | Yes | A | Cat | AR | [48] |
|  | No | No | No | Yes | No | No | R | Cat | HRP | [83] |
|  | No | No | No | Yes | No | Yes | A | Monkey^a^ | AR | [65] |
|  | N/A | N/A | N/A | Yes | N/A | N/A | A | Cat | AR | [217] |
|  | N/A | N/A | N/A | Yes | No | Yes | A | Rat | WGA-HRP | [218] |
|  | N/A | N/A | N/A | Yes | N/A | N/A | A | Cat | AR | [55] |
|  | N/A | N/A | N/A | Yes | N/A | N/A | R | Rabbit | HRP | [219] |
| **Red nucleus** | No | No | No | Yes | No | Yes | R | Rat | WGA-HRP | [220] |
|  | No | No | No | Yes | No | No | A | Cat | WGA-HRP | [231] |
|  | N/A | N/A | N/A | N/A | N/A | Yes | A | Cat | WGA-HRP | [232] |
|  | N/A | N/A | N/A | N/A | N/A | Yes | A | Rat | AR | [225] |
|  | No | No | No | Yes | No | Yes | R | Rat | WGA-HRP | [221] |
|  | N/A | N/A | N/A | Yes | N/A | N/A | A | Squirrel | WGA-HRP | [233] |
|  | N/A | N/A | N/A | Yes | N/A | N/A | R | Cat | WGA-HRP | [222] |
| **Red nucleus** | N/A | Yes | Yes | Yes | Yes | Yes | A | Rat | WGA-HRP | [234] |
|  | No | No | No | Yes | No | Yes | R | Rat | WGA-HRP, AR | [235] |
|  | No | No | No | Yes | No | Yes | R | Monkey^e^ | HRP | [79] |
|  | N/A | N/A | N/A | Yes | N/A | Yes | A | Rat | WGA-HRP | [85] |
|  | N/A | N/A | N/A | Yes | N/A | N/A | A | Monkey^b^ | HRP | [67] |
|  | N/A | N/A | N/A | Yes | N/A | N/A | A | Kitten | PHA-L | [223] |
|  | No | No | No | Yes | No | No | R | Rabbit | HRP | [236] |
| **Red nucleus** | N/A | Yes | Yes | Yes | Yes | Yes | A | Rat | WGA-HRP | [237] |
|  | No | No | No | Yes | No | Yes | R | Guinea pig | WGA:apoHRP-Au | [238] |
|  | No | No | No | Yes | No | Yes | R | Rabbit | HRP | [239] |
|  | N/A | Yes | Yes | Yes | Yes | Yes | A | Dog | WGA-HRP | [240] |
|  | N/A | N/A | N/A | Yes | N/A | Yes | A | Monkey | WGA-HRP | [241] |
|  | N/A | Yes | Yes | Yes | Yes | Yes | R | Rat | Ctb | [95] |
|  | N/A | N/A | N/A | N/A | N/A | Yes | A | Rat | BDA | [117] |
| **Red nucleus** | No | No | No | Yes | No | Yes | R | Rat | Ctb, WGA-HRP | [121] |
|  | No | No | No | Yes | No | Yes | A/R | Cat | WGA-HRP | [111] |
|  | No | No | No | Yes | No | No | R | Mouse | FG | [242] |
|  | N/A | Yes | Yes | Yes | Yes | Yes | A | Rat | BDA, PHA-L | [243] |
|  | No | Yes | Yes | Yes | Yes | Yes | A | Mouse | H129ΔTK-TT virus | [93] |
|  | No | Yes | No | Yes | No | Yes | R | Rat | Ctb, GL | [110] |
|  | No | No | No | Yes | No | Yes | R | Rat | WGA | [244] |
| **Red nucleus** | No | Yes | Yes | Yes | Yes | Yes | A | Mouse, cerebellar plate in embryonic development | DC | [245] |
|  | N/A | N/A | N/A | Yes | N/A | N/A | A | Mouse | AAV, BDA | [224] |
|  | No | Yes | Yes | Yes | Yes | Yes | A/R | Rat | BDA, FG | [64] |
|  | N/A | N/A | N/A | Yes^1^ | N/A | N/A | A | Mouse | AAV | [77] |
|  | N/A | Yes | Yes | Yes | Yes | Yes | A | Mouse | AAV | [34] |
|  | N/A | N/A | N/A | Yes^1^ | N/A | N/A | A | Mouse | Ctb | [11] |
|  | N/A | N/A | N/A | Yes | N/A | N/A | A | Mouse | AAV | [32] |
|  | | | | | | | | | | |
| **Ventral tegmental area** | No | Yes | No | No | No | No | R | Rat | HRP | [259] |
|  | No | No | No | No | No | Yes | R | Rat | HRP | [228] |
|  | No | No | No | Yes | No | Yes | A/R | Rat | WGA-HRP, HRP | [85] |
|  | No | No | No | No | No | Yes | R | Mouse | RV | [260] |
|  | N/A | N/A | N/A | N/A | N/A | Yes | A | Rat | PHA | [258] |
|  | N/A | Yes | Yes | Yes | Yes | Yes | A | Mouse | AAV | [37] |
|  | No | No | No | Yes | No | Yes | A/R | Mouse | AAV, rAAV | [257] |
|  | N/A | N/A | N/A | Yes | N/A | N/A | A | Mouse | AAV | [32] |
|  | | | | | | | | | | |
| **Mesodiencephalic junction** | N/A | Yes | N/A | N/A | N/A | N/A | A | Monkey | AR | [54] |
|  | No | No | No | Yes | No | Yes | A | Monkey^d^ | AR | [230] |
|  | No | Yes | Yes | Yes | Yes | Yes | A | Cat | AR | [48] |
|  | No | Yes | Yes | Yes | Yes | Yes | A | Cat | AR | [83] |
|  | N/A | Yes | N/A | Yes | N/A | Yes | A | Cat | AR | [207] |
|  | N/A | No | N/A | Yes | N/A | Yes | A | Monkey^a,d^ | AR | [365] |
|  | No | Yes | No | No | No | No | A | Monkey^a^ | AR | [65] |
|  | N/A | N/A | N/A | No | N/A | Yes | A | Rat | WGA-HRP | [218] |
|  | No | No | No | No | No | Yes | R | Cat | HRP, WGA-HRP, AR | [139] |
|  | No | Yes | No | No | No | Yes | R | Cat | HRP | [276] |
|  | No | Yes | No | No | No | No | R | Rat | TB | [173] |
|  | N/A | Yes | Yes | Yes | Yes | Yes | A | Rat | WGA-HRP | [234] |
|  | No | Yes | Yes | Yes | Yes | Yes | R | Monkey^e^ | HRP | [79] |
|  | N/A | N/A | N/A | Yes | N/A | Yes | A | Cat | WGA-HRP | [280] |
|  | No | Yes | No | No | No | No | A/R | Rat | WGA-HRP,  HRP | [275] |
|  | No | Yes | Yes | Yes | Yes | Yes | R | Rabbit | HRP | [273] |
|  | Yes | Yes | No | No | No | No | R | Monkey^f^ | FB, DY | [180] |
|  | No | No | No | No | No | No | A | Monkey | WGA-HRP | [279] |
|  | No | Yes | No | Yes | No | Yes | A/R | Rat | WGA-HRP, FG | [209] |
|  | N/A | Yes | N/A | Yes | N/A | Yes | A | Dog | WGA-HRP | [240] |
|  | N/A | N/A | N/A | Yes | N/A | N/A | A | Cat | WGA-HRP | [278] |
|  | N/A | N/A | N/A | Yes | N/A | Yes | A | Monkey^i^ | WGA-HRP | [281] |
|  | No | No | No | Yes | No | No | R | Rat | FG | [277] |
|  | No | Yes | No | Yes | Yes | Yes | R | Rat | Ctb, GL | [110] |
|  | No | Yes | Yes | Yes | Yes | Yes | R | Rat | BDA, FG | [64] |
|  | N/A | Yes | Yes | N/A | N/A | N/A | A | Mouse | AAV | [33] |
|  | N/A | Yes | N/A | Yes | N/A | Yes | A | Mouse | AAV | [34] |
|  | N/A | Yes | N/A | N/A | N/A | N/A | A/R | Monkey^l^ | BDA, RV | [274] |
|  | No | Yes | N/A | Yes | N/A | Yes | A/R | Mouse | Ctb, RV, AAV | [261] |
|  | | | | | | | | | | |
| **EW** | N/A | Yes | N/A | No^2^ | N/A | N/A | A | Monkey | WGA-HRP | [279] |
|  | No | No | No | Yes | No | No | R | Rat | FG | [277] |
|  | No | Yes | Yes | Yes | Yes | Yes | A/R | Rat | BDA, FG | [64] |
|  | N/A | Yes | N/A | Yes | N/A | Yes | A | Mouse | AAV | [34] |
|  | | | | | | | | | | |
| **3N** | No | No | No | Yes | No | Yes | A | Monkey^d^ | AR | [230] |
|  | N/A | No | N/A | Yes | N/A | Yes | A | Mouse | AAV | [34] |
|  | N/A | N/A | N/A | Yes | N/A | N/A | A | Mouse | AAV | [32] |
|  | | | | | | | | | | |
| **Superior colliculus** | N/A | Yes | N/A | N/A | N/A | N/A | A | Monkey | AR | [54] |
|  | No | Yes | No | Yes | No | Yes | R | Cat | HRP | [306] |
|  | No | Yes | No | Yes | No | Yes | R | Cat | HRP | [305] |
|  | No | Yes | No | Yes | Yes | Yes | R | Rat | TB, EB, DAPI | [44] |
|  | No | Yes | Yes | Yes^2^ | Yes | Yes | A/R | Cat | HRP, AR | [48] |
|  | No | Yes | No | Yes^2^ | No | Yes | A/R | Cat | HRP, AR | [83] |
|  | N/A | Yes | No | Yes^2^ | No | Yes | A | Cat | AR | [207] |
|  | N/A | Yes | No | No | No | No | A | Monkey^a^ | AR | [65] |
|  | No | No | No | Yes | No | Yes | R | Rabbit | HRP | [310] |
|  | N/A | Yes | N/A | N/A | N/A | N/A | A | Dog | AR | [49] |
|  | No | Yes | Yes | Yes | Yes | Yes | R | Rat | WGA-HRP | [302] |
|  | N/A | Yes | N/A | N/A | N/A | N/A | A | Dog | AR | [208] |
|  | No | Yes | No | Yes | No | Yes | A/R | Bat | WGA-HRP | [307] |
|  | No | Yes | No | Yes^2^ | No | Yes | R | Rat | TB | [173] |
|  | No | No | No | Yes | No | No | A/R | Squirrels | WGA-HRP, HRP | [233] |
|  | No | No | No | Yes^2^ | No | Yes | A/R | Rat | WGA-HRP | [311] |
|  | No | Yes | No | Yes^2^ | No | No | R | Monkey^e^ | HRP | [79] |
|  | No | Yes | No | Yes | No | Yes | R | Rat | WGA-HRP | [301] |
|  | No | Yes | No | Yes | No | yes | R | Rat | TB | [92] |
|  | N/A | N/A | N/A | Yes | N/A | N/A | A | Monkey ^b^ | HRP | [67] |
|  | N/A | Yes | N/A | N/A | N/A | N/A | A | Rat | WGA-HRP | [275] |
|  | N/A | Yes | Yes | Yes | Yes | Yes | A | Rat | WGA-HRP | [237] |
|  | No | Yes | Yes | Yes | Yes | Yes | A/R | Rat | WGA-HRP | [304] |
|  | No | Yes | Yes | Yes | Yes | Yes | R | Hedgehog | BDA, WGA-HRP, AR | [303] |
|  | No | Yes | No | No | No | No | R | Cat | FB | [309] |
|  | No | Yes | No | No | No | No | R | Cat | FB, NY | [308] |
|  | N/A | Yes | Yes | Yes | Yes | Yes | A | Rat | BDA, PHA-L | [243] |
|  | N/A | N/A | N/A | Yes^1^ | N/A | N/A | A | Mouse | AAV | [77] |
|  | N/A | N/A | N/A | Yes | N/A | Yes | A | Mouse | AAV | [294] |
|  | N/A | Yes | N/A | N/A | N/A | N/A | A | Mouse | AAV | [33] |
|  | N/A | N/A | N/A | Yes^1^ | N/A | N/A | A/R | Mouse | Ctb | [11] |
|  | N/A | N/A | N/A | Yes | N/A | N/A | A | Mouse | AAV | [32] |
|  | | | | | | | | | | |
| **Periaqueductal gray** | Not | Yes | No | Yes | No | Yes | R | Rat | HRP | [323] |
|  | No | Yes | No | No | No | No | A | Cat | AR | [48] |
|  | No | Yes | No | Yes^2^ | No | Yes | A | Cat | AR | [83] |
|  | No | Yes | No | No | No | No | A | Monkey^a^ | AR | [65] |
|  | N/A | Yes | N/A | N/A | N/A | N/A | A | Dog | AR | [208] |
|  | No | Yes | No | Yes | No | Yes | R | Rat | WGA-HRP | [301] |
|  | N/A | N/A | N/A | Yes | N/A | N/A | A | Monkey^b^ | HRP | [67] |
|  | N/A | Yes | N/A | N/A | N/A | N/A | A | Rat | WGA-HRP | [275] |
|  | No | Yes | Yes | Yes | Yes | Yes | A/R | Rat | BDA, FG | [64] |
|  | No | Yes | No | No | No | No | A/R | Mouse | AAV, rAAV, Ctb | [35] |
|  | N/A | Yes | Yes | N/A | N/A | N/A | A | Mouse | AAV | [33] |
|  | N/A | Yes | No | No | No | No | A | Mouse | AAV, Ctb or retrobeads | [38] |
|  | N/A | N/A | N/A | Yes | N/A | N/A | A | Mouse | AAV | [32] |
|  | | | | | | | | | | |
| **Pretectal complex** | No | Yes | No | Yes | No | Yes | R | Cat | FB | [485] |
|  | No | Yes | No | Yes | No | Yes | R | Cat | HRP | [48] |
|  | No | Yes | No | Yes | No | Yes | A/R | Cat | AR, HRP | [83] |
|  | N/A | No | No | Yes^1^ | No | Yes | A | Cat | AR | [207] |
|  | N/A | Yes | N/A | N/A | N/A | N/A | A | Dog | AR | [208] |
|  | No | Yes | No | Yes | No | Yes | R | Cat | WGA-HRP | [292] |
|  | N/A | Yes | N/A | Yes | N/A | Yes | A | Rat | WGA-HRP | [237] |
|  | N/A | N/A | N/A | Yes | N/A | N/A | A | Rat | DB, DT, DF | [295] |
|  | N/A | N/A | N/A | Yes | N/A | Yes | A | Rat | DB | [296] |
|  | No | Yes | Yes | Yes | Yes | Yes | R | Cat | BDA | [291] |
|  | No | Yes | No | No | No | Yes | R | Cat | WGA-HRP | [293] |
|  | N/A | Yes | N/A | Yes | N/A | Yes | A | Rat | BDA, PHA-L | [243] |
|  | No | Yes | Yes | Yes | Yes | Yes | A/R | Rat | BDA, FG | [64] |
|  | N/A | N/A | N/A | Yes | N/A | Yes | A | Mouse | AAV | [294] |
|  | No | Yes | No | Yes | No | Yes | R | Monkey^l^ | RV | [274] |
|  | N/A | N/A | N/A | Yes | N/A | N/A | A | Mouse | AAV | [32] |
|  | | | | | | | | | | |
| **DpMe** | No | No | No | No | No | Yes | R | Rat | HRP | [325] |
|  | N/A | Yes | No | N/A | N/A | N/A | A | Mouse | AAV | [33] |
|  | | | | | | | | | | |
| S**ubstantia nigra** | N/A | N/A | N/A | Yes^1^ | N/A | N/A | A | Cat | AR | [55] |
|  | N/A | N/A | N/A | Yes | N/A | Yes | A | Rat | WGA-HRP | [85] |
|  | No | No | No | No | No | Yes | R | Mouse | RV | [260] |
|  | No | Yes | Yes | Yes | Yes | Yes | A/R | Rat | BDA, FG | [64] |
|  | N/A | Yes | No | N/A | N/A | N/A | A | Mouse | AAV | [33] |
|  | | | | | | | | | | |
| **Hypothalamus** | N/A | N/A | N/A | Yes^1^ | N/A | Yes | A/R | Monkey^m^ | WGA-HRP | [42] |
|  | N/A | Yes | Yes | Yes | Yes | Yes | A | Monkey^c^ | WGA-HRP | [337] |
|  | N/A | Yes | Yes | Yes | Yes | Yes | A | Cat | WGA-HRP | [336] |
|  | N/A | N/A | N/A | Yes^2^ | N/A | Yes | A | Tree shrew | WGA-HRP | [342] |
|  | N/A | N/A | N/A | Yes^2^ | N/A | Yes | A | Monkey | WGA-HRP | [341] |
|  | N/A | N/A | N/A | Yes | N/A | N/A | A | Rat | FR | [340] |
|  | No | Yes | No | Yes | No | Yes | R | Rat | HRP | [338] |
|  | No | Yes | No | No | No | Yes | R | Rat | HRP | [343] |
|  | No | Yes | No | No | No | No | A/R | Rat | BDA, FG | [339] |
|  | N/A | Yes | No | N/A | N/A | N/A | A | Mouse | AAV | [33] |
|  | | | | | | | | | | |
| **Thalamus** | N/A | Yes  CM, VPM, VP-VL, VL, PC,  pf | N/A | N/A | N/A | N/A | A | Monkey | AR | [54] |
|  | No | No | No | Yes  VL | No | No | R | Cat | HRP | [105] |
|  | N/A | Yes  VM, VL, IL, CM, PC | Yes  VM, VL, IL, CM, PC | Yes  VM, VL, IL, CM, PC | Yes  VM, VL, IL, CM, PC | Yes  VM, VL, IL, CM, PC | A | Cat | AR | [227] |
|  | No | Yes  CM | No | No | No | No | A | Monkey^a^ | AR | [65] |
|  | N/A | Yes  VL, CL, VPL | N/A | Yes  VPI, VPL, VL, CL | N/A | Yes  VP,  VL, CL | A | Monkey^a,d^ | AR | [365] |
|  | N/A | Yes  Cat: VL, VPL  Monkey: VL, VPL, VPM, Vim | N/A | Yes  Cat: VL, VPL  Monkey: VL, VPL, VPM, Vim | N/A | Yes  Cat: VL, VPL  Monkey: VL, VPL, VPM, Vim | A | Cat, monkey | AR | [380] |
|  | N/A | N/A | N/A | No | N/A | Yes | A | Rat | WGA-HRP | [218] |
| **Thalamus** | No | Yes | No | No | No | No | R | Rat | FG | [130] |
|  | No | No | No | No | No | Yes  LP | R | Cat | HRP | [378] |
|  | No | Yes  VM | No | No | No | Yes  VM | R | Cat | HRP | [368] |
|  | No | Yes | No | Yes | No | Yes | R | Rat | FB, NY, DY | [45] |
|  | N/A | Yes  PVT, MD, CM, PC, pf, CL, VM, VL | N/A | N/A | N/A | N/A | A | Dog | AR | [208] |
|  | N/A | Yes  MD | Yes  MD | Yes  MD | Yes  MD | Yes  MD | R | Dog | HRP | [353] |
|  | N/A | No | No | Yes | No | No | A | Cat | WGA-HRP | [231] |
| **Thalamus** | No | Yes  VM, VA, VL | No | Yes  VM, VA, VL | No | Yes  VM, VA, VL, IL | R | Cat | HRP | [358] |
|  | N/A | Yes  CL | N/A | Yes^1^  VM, VL, VP-VL, CM, pf, CL, DM | N/A | Yes  VPL, VL, CL | A | Monkey^d^ | AR | [357] |
|  | No | Yes  VL, VM | No | Yes  VL, VM | No | Yes  VL, VM | R | Cat | HRP | [360] |
|  | No | Yes  VM, VL, CL, CM, PC | No | No | No | No | A/R | Cat | WGA-HRP | [367] |
|  | No | Yes  VL, CL | Yes  VL, CL | Yes  VL, CL | Yes  VL, CL | Yes  VL, CL | A/R | Rat | WGA-HRP, FB | [234] |
|  | No | Yes  pf | No | Yes  pf | No | No | R | Rat | WGA | [362] |
|  | No | Yes | No | Yes | No | Yes | R | Rat | TB, NY, WGA-HRP | [92] |
| **Thalamus** | N/A | N/A | N/A | Yes  VPL, VPLo, VL, CL, IL | N/A | N/A | A | Monkey^b^ | HRP | [67] |
|  | N/A | No | No | Yes | No | No | A | Monkey | WGA-HRP | [341] |
|  | No | Yes  CM, pf | No | Yes  CM, pf | No | Yes  CM, pf | R | Cat | HRP, WGA-HRP, rhodamine microspheres | [363] |
|  | N/A | Yes  VL, IL | N/A | Yes  VL, IL | N/A | Yes  VL, IL | A | Rat | PHA-L, WGA-HRP | [356] |
|  | N/A | Yes | N/A | Yes | N/A | Yes | A | Monkey^c^ | Biocytin | [175] |
|  | N/A | Yes  IL, ventral LG | N/A | Yes  IL, ventral LG | N/A | Yes  IL, ventral LG | A | Rat | WGA-HRP | [237] |
|  | N/A | Yes  VL, VA, VL, VM, MD, CM, CL, pf | N/A | Yes  VL, VA, VL, VM, MD, CM, CL, pf | N/A | Yes  VL, VA, VL, VM, MD, CM, CL, pf | A | Dog | WGA-HRP | [240] |
| **Thalamus** | No | Yes  VM | No | Yes  VM | No | Yes  VM | R | Cat | WGA-HRP | [361] |
|  | N/A | N/A | N/A | N/A | N/A | Yes  VL, pf, CM, IL | A | Rat | DB | [377] |
|  | N/A | N/A | N/A | Yes  pf, CL, CM, LP, VL | N/A | Yes  pf, CL, CM, LP, VL | A | Rat | DB | [296] |
|  | N/A | N/A | N/A | Yes  VL, VPL, pf | N/A | N/A | A | Rat | DT, DF, DB | [295] |
|  | N/A | N/A | N/A | N/A | N/A | Yes  VL. | A | Monkey^d^ | WGA-HRP | [376] |
|  | N/A | N/A | N/A | Yes  VP, VL, MD, CM, CL | N/A | Yes  VP, VL, MD, CM, CL | A | Monkey^i^ | WGA-HRP | [281] |
|  | N/A | N/A | N/A | Yes  VL | N/A | Yes  VL. | A | Cat | WGA-HRP | [359] |
| **Thalamus** | N/A | N/A | N/A | N/A | N/A | Yes  VM, VL | A | Rat | BDA | [117] |
|  | N/A | Yes  VA, LP | No | Yes  VM, VA, CL, CM, LP, PVT | No | Yes  VM, VA, CL, CM, LP, PVT | A | Hedgehog | WGA-HRP, BDA | [104] |
|  | N/A | N/A | N/A | N/A | N/A | Yes  CL, VL, VM, PC, pf | A | Rat | BDA | [374] |
|  | No | No | No | Yes  RTN | No | Yes  RTN | R | Rat | HRP | [372] |
|  | N/A | N/A | N/A | Yes  VL. | N/A | N/A | A | Mouse, in vitro | BDA | [486] |
|  | N/A | No | No | Yes  VL | No | Yes  VL. | A | Monkey^g^ | BDA | [371] |
|  | No | Yes | Yes | Yes | Yes | Yes | R | Monkey^a^ | Ctb | [381] |
| **Thalamus** | No | No | No | No | No | Yes | R | Monkey^h^ | RV | [373] |
|  | No | Yes | No | Yes | No | Yes | R | Monkey^g^ | RV | [354] |
|  | No | Yes  VL | No | Yes  VL | No | Yes  VL | R | Monkey^a^ | Ctb | [355] |
|  | No | Yes | No | No | No | No | R | Mouse | FD | [52] |
|  | No | No | No | Yes | No | Yes | R | Rat | FG | [370] |
|  | N/A | Yes  VA, VL, Po, LD, pf | Yes  VA, VL, Po, LD, pf | Yes  VA, VL, Po, LD, pf | Yes  VA, VL, Po, LD, pf | Yes  VA, VL, Po, LD, pf | A | Rat | BDA, PHA-L | [243] |
|  | No | Yes  VL | Yes  VL | Yes  VL | Yes  VL | Yes  VL | A | Mouse | Cre-dependent H129ΔTK-TT virus | [93] |
| **Th** | No | No | No | No | No | Yes  MD | R | Rat | FG | [379] |
|  | No | Yes  Cerebellum to VL, VA VM, LP, Po, CM, PC, pf | Yes | Yes | Yes | Yes | A/R | Rat | BDA, FG | [64] |
|  | N/A | N/A | N/A | Yes^1^  VA, VL | N/A | N/A | A | Mouse | AAV | [77] |
|  | No | No | No | Yes^1^  LG | No | Yes  LG, RTN | A/R | Cat | WGA-HRP | [369] |
|  | N/A | N/A | N/A | Yes  Pom | N/A | Yes  Pom | A | Mouse | AAV | [294] |
|  | No | No | No | No | No | Yes  CL. | R | Mouse | LV | [375] |
|  | N/A | Yes  pf, MD, VM, VAL | N/A | N/A | N/A | N/A | A/R | Mouse | AAV, rAAV | [35] |
| **Thalamus** | N/A | Yes  MD, VM, VAL, VL, IL, CL, pf | Yes  VL | N/A | N/A | N/A | A | Mouse | AAV | [33] |
|  | N/A | Yes  CL, VM | N/A | Yes  VAL | N/A | Yes  VM, VAL | A | Mouse | AAV | [34] |
|  | N/A | N/A | N/A | Yes^1^  VL. | N/A | N/A | A | Mouse | Ctb | [11] |
|  | N/A | N/A | N/A | Yes  VAL, pf, VM, CM, PC, VPM | N/A | N/A | A | Mouse | AAV | [32] |
|  | No | Yes  VM, VAL, VPM, VPL, RTN | N/A | Yes  VM, VAL, VPM, VPL, RTN | N/A | Yes  VM, VAL, VPM, VPL, RTN | A | Mouse | Herpes simplex virus, rAAV | [148] |
|  | | | | | | | | | | |
| **Sth** | No | Yes | No | Yes | No | Yes | A | Cat | AR | [227] |
|  | No | No | No | Yes | No | Yes | A | Hedgehog | WGA-HRP, BDA | [104] |
| **Sth** | No | Yes | No | Yes | No | Yes | R | Rat | FG | [387] |
|  | No | Yes | Yes | Yes | Yes | Yes | A/R | Rat | BDA, FG | [64] |
|  | | | | | | | | | | |
| **Zona incerta** | No | Yes | No | Yes | No | Yes | A | Cat | AR | [227] |
|  | N/A | Yes | No | Yes^2^ | No | No | A | Cat | AR | [366] |
|  | No | No | No | Yes | No | No | A | Cat | AR | [48] |
|  | N/A | No | N/A | Yes | N/A | Yes | A | Monkey^a,d^ | AR | [365] |
|  | N/A | N/A | N/A | No | N/A | Yes | A/R | Rat | WGA-HRP | [218] |
|  | No | No | No | Yes | No | Yes | A/R | Rat | WGA-HRP, HRP | [395] |
|  | N/A | Yes | Yes | Yes | Yes | Yes | A | Rat | WGA-HRP | [234] |
|  | No | No | No | Yes | No | Yes | R | Cat | HRP | [394] |
|  | N/A | N/A | N/A | Yes | N/A | N/A | A | Monkey^b^ | HRP | [67] |
|  | N/A | Yes | Yes | Yes | Yes | Yes | A | Rat | WGA-HRP | [237] |
|  | N/A | Yes | Yes | Yes | Yes | Yes | A | Dog | WGA-HRP | [240] |
|  | N/A | N/A | N/A | N/A | N/A | Yes | A | Rat | DB | [377] |
|  | N/A | N/A | N/A | Yes | N/A | Yes | A | Rat | DB | [296] |
|  | N/A | N/A | N/A | Yes | N/A | N/A | A | Rat | DT, DF, DB | [295] |
|  | N/A | N/A | N/A | Yes | N/A | Yes | A | Monkey^i^ | WGA-HRP | [281] |
|  | N/A | No | No | Yes | No | Yes | A | Hedgehog | WGA-HRP, BDA | [104] |
|  | No | No | No | Yes | No | No | A/R | Rat | Ctb | [396] |
|  | No | No | No | Yes | No | Yes | R | Cat | WGA-HRP | [293] |
|  | N/A | Yes | Yes | Yes | Yes | Yes | A | Rat | BDA, PHA | [243] |
|  | No | Yes | Yes | Yes | Yes | Yes | A/R | Rat | BDA, FG | [64] |
|  | No | No | No | Yes | No | Yes | A | Mouse | AAV | [294] |
|  | N/A | Yes | No | N/A | N/A | N/A | A | Mouse | AAV | [33] |
|  | N/A | No | N/A | No | N/A | Yes | A | Mouse | AAV | [34] |
|  | N/A | N/A | N/A | Yes | N/A | N/A | A | Mouse | AAV | [32] |

^a^: Cynomolgus monkey.

^b^: Capuchin monkey.

^c^: Squirrel monkey.

^d^: Rhesus monkey.

^e^: New world monkey.

^f^: Pig-tailed monkey.

^g^: Macaque monkey.

^h^: Cebus monkey.

^i^: Japanese macaque.

^l^: Crab-eating macaque.

^m^: Prosimian primate.

^1^: IntA.

^2^: IntP.

N/A: not assessed; *T-area: transition area between the medial portions of the ventral lamella of the PO and the ventral fold of DAO.

**Structures:** 3N, oculomotor nucleus; 5N, trigeminal nucleus; 6N, abducens nucleus; 7N, facial nucleus; β, nucleus β of the inferior olive; Amb, Nucleus ambiguus; CL, centrolateral nucleus; CM, centromedial nucleus; CN, cerebellar nuclei; DAO, dorsal accessory olive; Dk, nucleus of Darkschewitsch; dmcl, dorsomedial cell group; DN, dentate nucleus; DpMe, deep mesencephalic nucleus; EW, Edinger-Westphal nucleus; FN, fastigial nucleus; Gi, gigantocellular nucleus; IL, intralaminar nucleus; InC, interstitial nucleus of Cajal; Int, interposed nucleus; IO, inferior olive; Kooy, dorsal cap of Kooy of the inferior olive; LD, latero-dorsal nucleus; LG, lateral geniculate nucleus; LP, latero-posterior nucleus; LVN, lateral vestibular nucleus; MAO, medial accessory olive; MD, mediodorsal thalamic nucleus; MG, medial geniculate nucleus; mRN, magnocellular part of red nucleus; mRtn, magnocellular reticular nucleus; MVN, medial vestibular nucleus; PAG, periaqueductal gray; PC, paracentral nucleus; PCs, Purkinje cells; pf, parafascicular complex; PGi, paragigantocellular nucleus; PMf, paramedian pontine reticular formation; Po, posterior thalamic nuclei; PO, principal olive; Pom, posterior medial nucleus; Pr, perihypoglossal nucleus; pRN, parvocellular part of red nucleus; PVT, paraventricular nucleus; RN, red nucleus; RTN, reticular thalamic nuclei; SpVN, spinal vestibular nucleus; Sth, subthalamic nucleus; SVN, superior vestibular nucleus; Tg, tegmentum; Th, thalamus; VA, ventro-anterior thalamic nucleus; VAL, ventro-antero thalamic complex; Vim, nucleus ventralis intermedius; VL, ventro-lateral thalamic nucleus; vlp, ventrolateral protuberance; VM, ventromedial nucleus; VN, vestibular nuclei; VPL, ventro-posterior lateral thalamic nucleus; VPM, ventro-posterior medial thalamic nucleus; VRG, ventral respiratory group.

**Tracers:** AAV: adeno-associated virus; AR: Autoradiographic method; BB: Bisbenzimide; BDA: Biotinylated dextran amines; CAV: Canine adenovirus; DY: Diamidino yellow; DF. Dextran-Fluoroscein; DT: Dextran-tetramethylrhodamine; DB: Dextran-biotin; DC: Diakylcarbocyanines; EB: Evans blue; FB: Fast blue; FD: Fluorescent dextran; FG: Fluoro gold; FR: Fluoro-ruby; GB: Granular blue; GL: Gold lectin; HRP: Horse radish peroxidase; LV: Lentivirus; NY: Nuclear yellow; TB: True blue; PHA-L: Phaseolus vulgaris leucoagglutinin; RV: Rabies virus; rAAV: Retrograde adeno-associated virus; WGA-HRP: Wheat germ conjugated with horse radish peroxidase; WGA-BSA-Gold: Gold-lectin conjugate with WGA , bovine serum albumin; WGA:apoHRP-Au: Colloidal gold-labeled enzymatically inactive WGA-HRP

**Table S3 Fractional anisotropy (FA) values of the superior cerebellar peduncle (SCP) in patients affected by ataxia, schizophrenia, and autism.**

| **Reference** | **Disease** | **Method** | **Sample** | **SCP FA values** |
| --- | --- | --- | --- | --- |
| [450] | SCA 1 | MRI and DTI | 6 Ataxia patients.  8 healthy controls. | SCA1: 0.41.  Control: left, 0.53; right, 0.52.  Average control: 0,53. |
| [451] | SCA 1 and SCA2 | MRI and DTI | 14 SCA1 patients.  11 SCA2 patients.  9 healthy controls. | SCA1: 0.42.  SCA2: SCP: 0.43.  Control: 0.48. |
| [453] | SCA 3 | MRI and DTI | 22 Ataxia patients.  24 healthy controls. | SCA3: right, 0.58; left, 0.61.  Control: right, 0.70; left, 0.73. |
| [452] | SCA 1, 2, 3, | MRI and DTI | 11 SCA 1 patients.  9 SCA 2 patients.  7 SCA 3 patients.  9 healthy controls. | SCA1: right, 0.52; left, 0.522.  SCA2: right, 0.505; left, 0.508.  SCA3: right, 0.518; left, 0.502.  Control: right: 0.651; left: 0.638. |
| [456] | Friedreich's ataxia | MRI, DTI | 12 Ataxia patients.  14 healthy controls. | DN-RN tract: 0.24. Control: 0.36.  DN-Th tract: 0.26. Control: 0.40. |
| [455] | Friedreich's ataxia | MRI and DTI | 21 Ataxia patients.  17 healthy controls. | Friedreich's ataxia: 0.57.  Control: 0.66 |
|  | | | | |
| [459] | Schizophrenia | rs-fMRI and DTI | 10 schizophrenia patients.  10 healthy controls. | SZ: 0.615  Control: 0.700. |
| [460] | Schizophrenia | structural MR and DTI. | 12 patients with early-stage schizophrenia.  10 healthy controls. | SZ: left cerebellum to right thalamus, 0.588. Control, 0.616.  Right cerebellum to left thalamus, 0.538. Control, 0.593. |
| [461] | Schizophrenia | MRI, DTI | 21 schizophrenia patients.  21 healthy control. | SZ: right, 0.52. Control: 0.57.  Left 0.54. Control, 0.57. |
|  | | | | |
| [470] | Autism spectrum disorder (ASD) | MRI, DTI, Q-ball imaging (QBI) tractography | 13 children with high functioning ASD (HFA).  11 children with low functioning ASD (LFA).  14 healthy controls. | ASD: Dentatorubrothalamic tract from dorso rostral dentate nucleus right:  High- functioning autism (HFA), 0.443.  Low- functioning autism (LFA), 0.431.  Control, 0.508.  Dentatorubrothalamic tract from dorso caudal dentate nucleus: left, LFA, 0,314. Control, 0,4.  Right, HFA, 0,416. LFA, 0,396. Control, 0,455.  Dentatorubrothalamic tract from ventro-rostral dentate nucleus right: HFA, 0.445. LFA: 0.428. Control, 0.485.  Dentatorubrothalamic tract from ventro-caudal right: HFA, 0.418. LFA, 0.405. Control, 0.454. |
| [471] | Autism spectrum disorder (ASD) | MRI and DTI | 13 ASD patients.  11 healthy controls. | ASD: right, 0.43. Left, 0.44.  Control: right, 0.48. Left, 0.50. |
| [472] | Asperger syndrome | MRI, DTI | 15 Asperger syndrome patients.  16 healthy controls. | ASD: right, 0.45. Control: 0.52. |

**Structures**: 3N, Oculomotor nucleus; 5N, Trigeminal nucleus; 7N, Facial nucleus; 12N, Hypoglossal nucleus; DmTg, Dorsomedial tegmental area; DR, Dorsal raphe nuclei; DpMe, Deep mesencephalic nucleus; EW, Edinger-Westphal nucleus; Gi, Gigantocellular nucleus; Hyp, Hypothalamus; I5, Intertrigeminal nucleus; IO, Inferior olive; IP, Interpeduncular nucleus; IRt, Intermediate reticular nucleus; LDTg, Laterodorsal tegmental nucleus; LC, Locus Coeruleus; lPAG, Lateral periaqueductal gray; LRt, Lateral reticular nucleus; LVN, Lateral vestibular nucleus; M5N, Midbrain trigeminal nucleus; Mdf, Medullary reticular formation; MDJ, Mesodiencephalic junction; Mdn, Medullary reticular nucleus; Mo5, Motor trigeminal nucleus; MRn, Midbrain reticular nucleus; mRtn, Magnocellular reticular nucleus; MSRF, Mesencephalic reticular formation; MVN, Medial vestibular nucleus; PAG, Periaqueductal gray; PB, Parabrachial complex; PCom, Nucleus of the posterior commissure; PCRt, Parvocellular reticular nuclei; PDTg, Posterodorsal tegmental nucleus; PGi, Paragigantocellular nucleus; PMf, Paramedian pontine reticular formation; PN, Pontine nuclei; PPtg, Peduncolopontine tegmentum nucleus; Pr, Perihypoglossal nucleus; PRP, Prepositus hypoglossi nucleus; PSol, Parasolitary nucleus; PT, Pretectal complex; RMg, Magnus raphe nuclei; RNm, Red nucleus magnocellular part; RNp, Red nucleus parvicellular part; ROb, Obscurus raphe nuclei; RtTg, Nucleus reticularis tegmenti pontis; SN, Substantia nigra; Sol, Nucleus of the solitary tract; SC, Superior colliculus; SpVN, Spinal vestibular nucleus; Su3, Supraoculomotor periaqueductal gray; SVN, Superior vestibular nucleus; Tgn, Tegmental reticular nucleus; Th, Thalamus; vlPAG, Ventrolateral periaqueductal gray; VTA, Ventral tegmental area; VTn, Ventral tegmental nucleus; VTr, Ventral tegmental relay zone; ZI, Zona incerta.
